# Paeoniflorin mitigates insulin-like growth factor 1-induced lipogenesis and inflammation in human sebocytes by inhibiting the PI3K/Akt/FoxO1 and JAK2/STAT3 signaling pathways

**DOI:** 10.1007/s13659-024-00478-4

**Published:** 2024-10-01

**Authors:** Chuanchuan Cai, Si Liu, Yufeng Liu, Shaobin Huang, Shiya Lu, Fang Liu, Xiaohua Luo, Christos C. Zouboulis, Ge Shi

**Affiliations:** 1 Department of Dermatology, Affiliated Hospital of Guangdong Medical University, Zhanjiang, 524001 China; 2 Department of Cosmetic and Plastic Surgery, the Sixth Affiliated Hospital, Sun Yat-sen University, Guangzhou, 510655 China; 3 Biomedical Innovation Center, the Sixth Affiliated Hospital, Sun Yat-sen University, Guangzhou, 510655 China; 4 Huamei-Bond International College, Guangzhou, 510520 China; 5 Departments of Dermatology, Venereology, Allergology and Immunology, Staedtisches Klinikum Dessau, Brandenburg Medical School Theodor Fontane and Faculty of Health Sciences Brandenburg, 06847 Dessau, Germany

**Keywords:** Paeoniflorin, Acne vulgaris, Sebocytes, Insulin-like growth factor-1

## Abstract

Insulin-like growth factor-1 (IGF-1) is considered as a pathogenic factor contributing to sebaceous gland dysfunction, which leads to acne vulgaris. Paeoniflorin (Pae), a bioactive monomer derived from total glycosides of paeony, has shown potential in treating various diseases. However, its anti-acne effects on human sebocytes are not well understood. In this study, we investigated the effects of Pae on acne development induced by IGF-1 in SZ95 sebocytes. Following IGF-1 stimulation, SZ95 sebocytes were exposed to Pae and then determined for proliferation, cell cycle, apoptosis, lipogenesis and pro-inflammatory cytokine secretion. We also analyzed the expression of proteins involved in the PI3K/Akt/FoxO1 and JAK2/STAT3 pathways. In vitro experiments demonstrated that Pae significantly inhibited colony formation, induced G1/S cell cycle arrest, promoted apoptosis, inhibited lipogenesis and cytokine synthesis in IGF-1-treated SZ95 sebocytes. Furthermore, Pae suppressed the phosphorylation of Akt, FoxO1, JAK2, and STAT3. Importantly, the sebo-suppressive and anti-inflammatory effects of Pae were enhanced by blocking PI3K and JAK2. In summary, our findings suggest that Pae has potent anti-proliferative and pro-apoptotic effects in SZ95 sebocytes. Additionally, Pae effectively protects against IGF-1-induced lipogenesis and inflammation by targeting the PI3K/Akt/FoxO1 and JAK2/STAT3 signaling pathways.

**Graphical Abstract:**

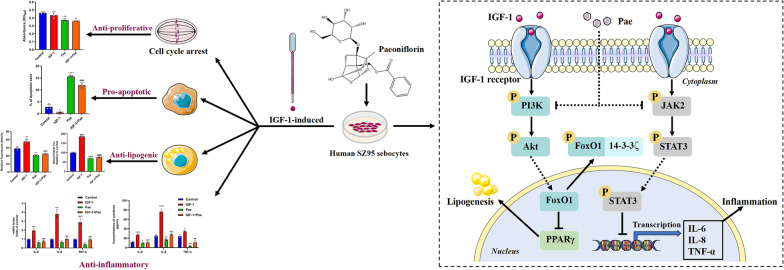

**Supplementary Information:**

The online version contains supplementary material available at 10.1007/s13659-024-00478-4.

## Introduction

Acne vulgaris (acne) is the most prevalent dermatosis that has become a significant health concern worldwide. Epidemiological estimates indicate that teenage acne is occurring at an earlier age, and the incidence of adult acne, particularly in females over 25 years old, is increasing [1, 2]. While oral isotretinoin is the only pharmacological intervention known to effectively treat moderate to severe acne, robust evidence has confirmed its embryotoxic, teratogenic, and potentially harmful properties [3].

The increased activity of sebaceous glands (SGs) plays a central role in the development of acne. Insulin-like growth factor (IGF)-1, a member of the insulin-related polypeptide family, is considered as a pivotal trigger in the pathogenesis and progression of acne. During puberty, the surge in growth hormone secretion stimulates hepatic synthesis of IGF-1 [4]. Western diets (WDs), characterized by high carbohydrate content and a high insulin index, have been proposed as exogenous risk factors for acne. WDs can elevate the circulating levels of both insulin and IGF-1, which subsequently activates the phosphoinositide 3-kinase/protein kinase B (PI3K/Akt) signaling pathway [5, 6]. In nutrient-sensing acne, IGF-1 promotes the nuclear export of forkhead box O1 (FoxO1) by phosphorylating PI3K and Akt, leading to sebum overproduction and altered lipid profile [7]. Therefore, pharmacological attenuation of IGF-1-mediated intracellular transduction may hold promise for the therapeutic management of acne.

In the Janus kinase (JAK) family, activated JAK2 induces tyrosine phosphorylation, providing a docking site for the recruitment of the signal transducer and activator of transcription 3 (STAT3) protein [8]. Inhibiting JAK prevents the phosphorylation of STAT3, thereby blocking IL-6-mediated inflammatory trans-signaling in human vascular endothelial cells [9]. Leptin, a pro-inflammatory hormone/cytokine, was found to enhance STAT3 phosphorylation, thus modulating the secretion of IL-6 and IL-8 in SZ95 sebocytes [10]. Additionally, IGF‑1 signaling has been shown to induce the inflammation associated with the aging process in a STAT3-dependent manner [11]. However, the relationship between of IGF-1 and the JAK2/STAT3 signaling pathway in sebaceous inflammation still remains uncertain.

*Paeonia lactiflora Pallas* (PLP) has been extensively utilized in China for over a millennium to treat autoimmune disorders [12]. The significant medicinal value of PLP has been demonstrated in chronic inflammatory skin conditions, such as atopic dermatitis [13]. Furthermore, its potential as a cosmetic product for enhancing the skin barrier function has been established [14]. Total glycosides of paeony (TGP) is the main bioactive substance extracted from the root of PLP. Among the monoterpene glycosides identified from TGP, paeoniflorin (Pae) stands out as the predominant constituent, accounting for over 40% of TGP [12]. Recent studies have elucidated that paeoniflorin exerts diverse protective and therapeutic effects by inhibiting the activated transduction of the PI3K/Akt and JAK2/STAT3 signaling pathway [15, 16].

In this study, we investigated the effects and molecular mechanisms of paeoniflorin in SZ95 sebocytes under IGF-1 stimulation. Our results revealed that paeoniflorin suppressed cell proliferation, induced cell cycle arrest and apoptosis in IGF-1-treated SZ95 sebocytes. Additionally, paeoniflorin ameliorated IGF-1-induced lipogenesis and inflammation by inhibiting the PI3K/Akt and JAK2/STAT3 signaling pathways. These data suggest that paeoniflorin could be a promising candidate for acne therapy.

## Results and discussion

### Effects of Pae on cell viability of SZ95 sebocytes

**Fig. 1:**
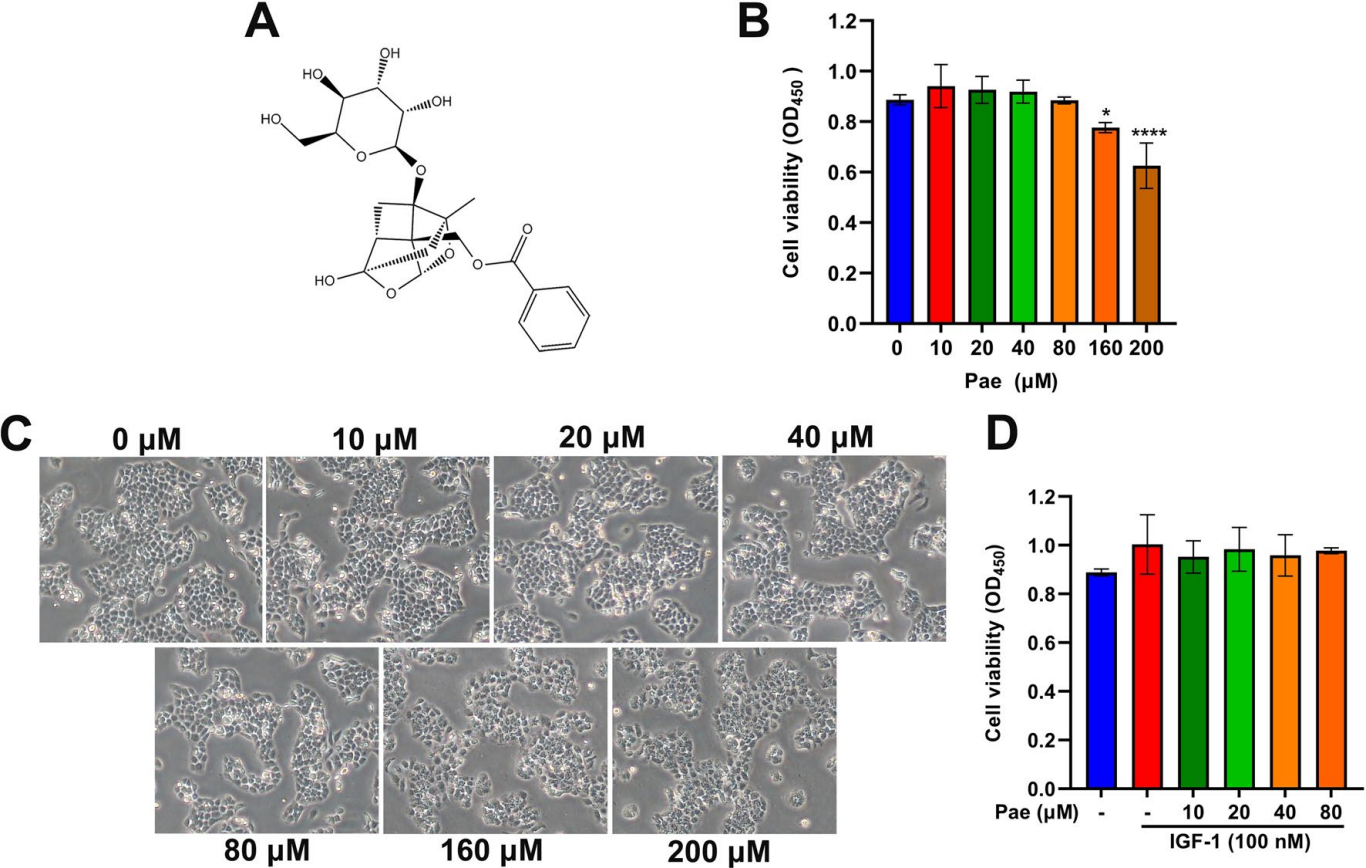
Effects of Pae on cell viability of SZ95 sebocytes. **A** Chemical structure of Pae. **B** Various doses of Pae (0, 10, 20, 40, 80, 160 and 200 µM) were applied to SZ95 sebocytes for 6 h. Cell viability was determined by CCK-8. **C** Representative morphology of cells with or without Pae treatment after IGF-1 stimulus was observed under microscopy. Magnification, × 200. **D** Cells were incubated with 10, 20, 40 and 80 µM Pae alone or in combination with 100 nM IGF-1, followed by CCK-8 determination. Data are shown as means ± SD, *n* = 6. *P < 0.05, ****P < 0.0001 vs. untreated cells

We first evaluated the cytotoxicity of Pae in cultured SZ95 sebocytes using Cell Counting Kit-8 (CCK-8) assay. By comparing the cell viability of untreated cells, we found that Pae at low concentrations (≤ 80 µM) for 6 h treatment exerted no cytotoxic effect on SZ95 sebocytes** (**Fig. 1B). Consistent with CCK-8 results, Pae did not cause significant changes in cellular morphology at low treatment concentrations (≤ 80 µM). When SZ95 sebocytes were exposed to high concentrations (≥ 160 µM), pronounced cellular disruption was evident, manifested by cell shrinkage, disorganized intracellular contents, and compromised intercellular contact regions (Fig. 1C). Furthermore, our findings showed that IGF-1 alone or in combination with Pae at doses up to 80 µM did not caused any cytotoxicity on SZ95 sebocytes (Fig. 1D). As a result, the optimal concentrations of 80 µM Pae and 100 nM IGF-1 were chosen for further experiments in SZ95 sebocytes.

### Pae suppressed cell proliferation and altered the phase distribution of cell cycle in SZ95 sebocytes

**Fig. 2:**
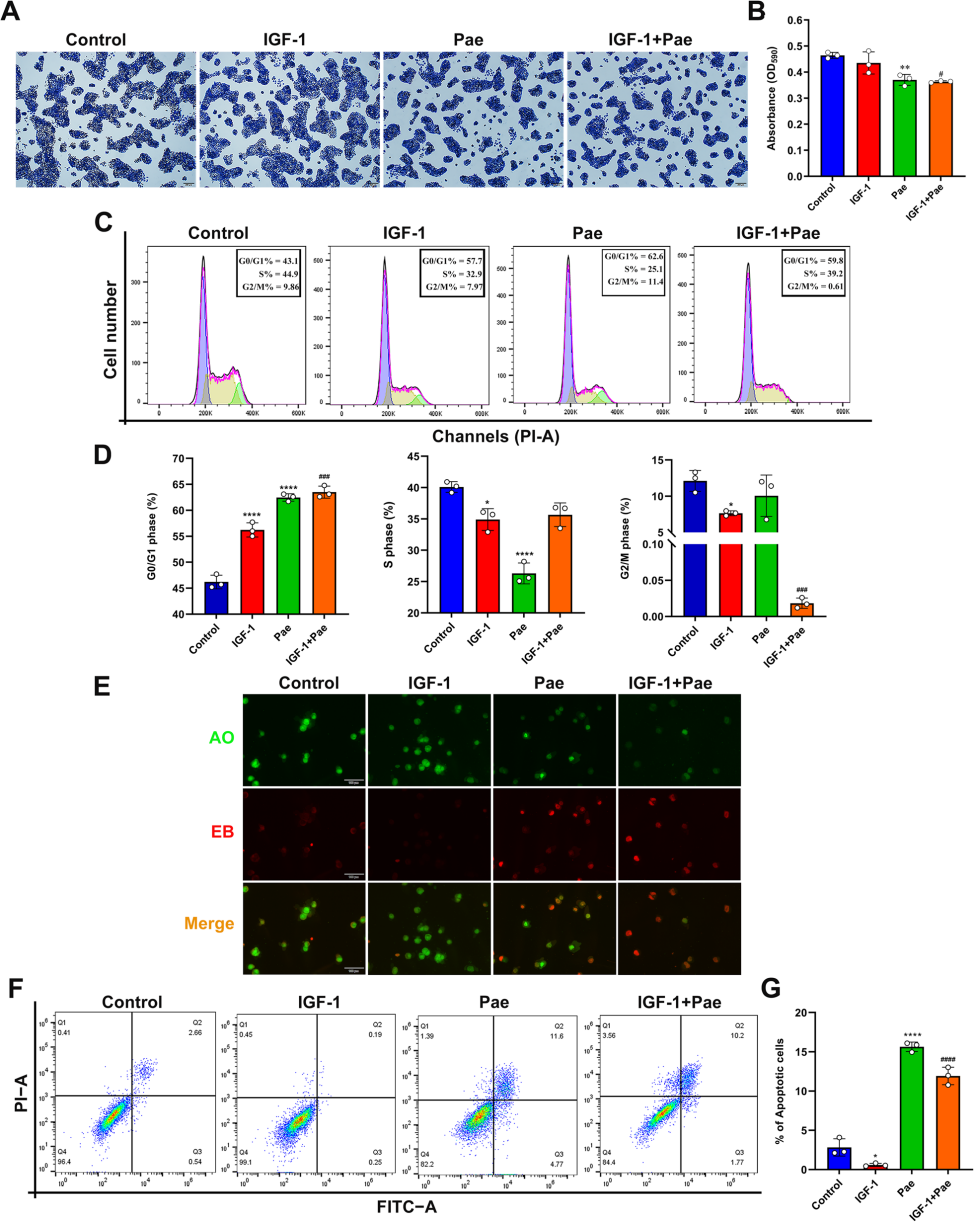
Effects of Pae on proliferation, cell cycle distribution and apoptosis in IGF-1-treated SZ95 sebocytes. After exposure to 100 nM IGF-1 for 1 h, cells were treated with 80 µM Pae for 6 h and then incubated in fresh medium for 24 h. **A** Cell proliferation was determined by crystal violet staining. Scale bars = 200 μm. **B **The optical density (OD) values of crystal violet were measured using a microplate reader at 590 nm. **C** Cell cycle progression was detected by flow cytometry using the PI/ RNase assay. G0/G1, interphase for DNA synthesis. S, DNA synthesis phase. G2/M, mitosis phase. **D** Cell cycle phase distribution (%) in SZ95 sebocytes treated with IGF-1 or Pae. **E** Representative morphology of apoptotic cells determined by AO/EB staining. Scale bars = 100 μm. **F** Cell apoptosis was assessed using the Annexin V/FITC assay. **G **The proportions of apoptosis include early and late apoptotic sebocytes. Data were expressed as mean ± SD, *n* = 3. *P < 0.05, **P < 0.01, ****P < 0.0001 vs. untreated cells. ^#^*P* < 0.05, ^###^*P* < 0.001, ^####^*P* < 0.0001 vs. IGF-1-treated cells

Following treatment with Pae, the colony formation of SZ95 sebocytes was detected by crystal violet assay. Microscopy revealed a notable reduction in cell proliferation in response to treatment with 80 µM Pae. The proliferative levels did not show a statistical significance between the untreated and IGF-1-treated cells, while Pae significantly suppressed cell proliferation following stimulation with 100 nM IGF-1 (Fig. 2A and B). To evaluate whether Pae induced inhibition of proliferation through cell cycle progression, flow cytometric analysis was performed. Our results indicated that Pae led to an increase in the proportion of cells in the G0/G1 phase and a decrease in the proportion of cells in the S phase, while the phase distribution in G2/M remained unaffected by Pae treatment. These findings demonstrate that Pae could impede cell cycle progression by inducing cell arrest in the G0/G1 phase, thereby hindering the transition from G1 to the S phase in SZ95 sebocytes. Treatment with IGF-1 alone increased the cell population at G0/G1 phase, while reducing the proportion in the S and G2/M phases compared to untreated cells. Interestingly, Pae decreased the cell cycle distribution at the G2/M phase in IGF-1-treated SZ95 sebocytes, indicating a collaborative effect of IGF-1 and Pae in inducing cell cycle arrest (Fig. 2C and D).

In acne lesions, the augmentation of proliferative processes in sebaceous gland cells leads to an increase in the total cell population, thereby resulting in a corresponding rise in the quantity of lipids synthesized [17]. IGF-1 is considered as a mitogen that is essential for the proliferation and differentiation of epidermal cells. However, there is still controversy regarding the relationship between IGF-1 and sebocyte proliferation in vitro. Insulin and IGF-1 have been found to increase cell proliferation in rat preputial sebocytes [18]. Exposure to 1 µM insulin for 24 h stimulated the growth and proliferation of sebocytes, while another study conducted by Mirdamadi et al. [7]. reported that treatment with 1 µM insulin and 0.1 µM IGF-1 for 24 h resulted in a decrease in DNA replication in cultured SZ95 sebocytes. In this study, we found that the colony formation of SZ95 sebocytes was not affected compared to untreated cells after treatment with 100 nM IGF-1 for 24 h. These findings suggest that the effects of insulin and IGF-1 on sebocyte growth and replication may vary depending on the specific experimental conditions and cell models used.

However, flow-cytometric results indicated that IGF-1 blocked the phase transition from G0/G1 to S, suggesting that IGF-1 could prevent cells from entering the mitotic process necessary for DNA synthesis. Therefore, we hypothesized that although IGF-1 may hinder sebocyte proliferation by arresting cell cycle distribution, it required time for these effects to be reflected on the cellular proliferative processes.

### Pae induced cell apoptosis in IGF-1-treated SZ95 seboctyes

To investigate the regulatory role of Pae in cell survival, the apoptotic process in SZ95 sebocytes was examined using the AO/EB staining kit. The AO dye labels the nuclei with green fluorescence, while EB only diffuses into cells with compromised membrane emitting red fluorescence. Cells treated with IGF-1 exhibited intense green fluorescence in the nuclei, with only a few apoptotic nuclei showing red fluorescence compared to untreated cells (Fig. 2E). In contrast, cells treated with Pae displayed early-stage apoptotic characteristics with pyknotic green nuclear staining and late-stage apoptotic cells with irregularly condensed orange-red nuclear staining. Apoptotic events were further assessed using Annexin V-FITC/PI staining. Our results indicated that IGF-1 reduced the proportion of apoptotic cells compared to untreated cells. In contrast, pre-incubation with Pae induced a significant increase of the apoptotic events following IGF-1 stimulation compared to the group treated with IGF-1 alone (Fig. 2F and G). Therefore, these findings suggest that Pae can reverse the inhibitory effect of IGF-1 on apoptosis in SZ95 sebocytes.

Apoptosis is a programmed cell death process under physiological conditions, which does not disrupt intracellular contents, thereby preventing the release of inflammatory mediators that could lead to tissue injury [19]. Isotretinoin, the most effective anti-acne agent, exhibits the strongest sebum-suppressive effect by inducing sebocyte apoptosis [3]. Therefore, promoting sebocyte apoptosis can not only reduce sebum secretion but also prevent excessive inflammatory responses. Consistent with previous studies [20], we found that IGF-1 exerted an inhibitory effect on apoptosis in SZ95 sebocytes. Pae has been proven to inhibit tumorigenesis by suppressing proliferation and inducting apoptosis in cancer cells [21]. Also, we found that Pae inhibited proliferation, arrested cell cycle progression and induced apoptosis in IGF-1-treated SZ95 sebocytes, suggesting that Pae has protective effects against IGF-I-induced sebocyte dysfunction with respect to cell growth and survival.

### Pae ameliorated IGF-1-induced lipogenesis and downregulated lipogenic transcription factors in SZ95 sebocytes

**Fig. 3:**
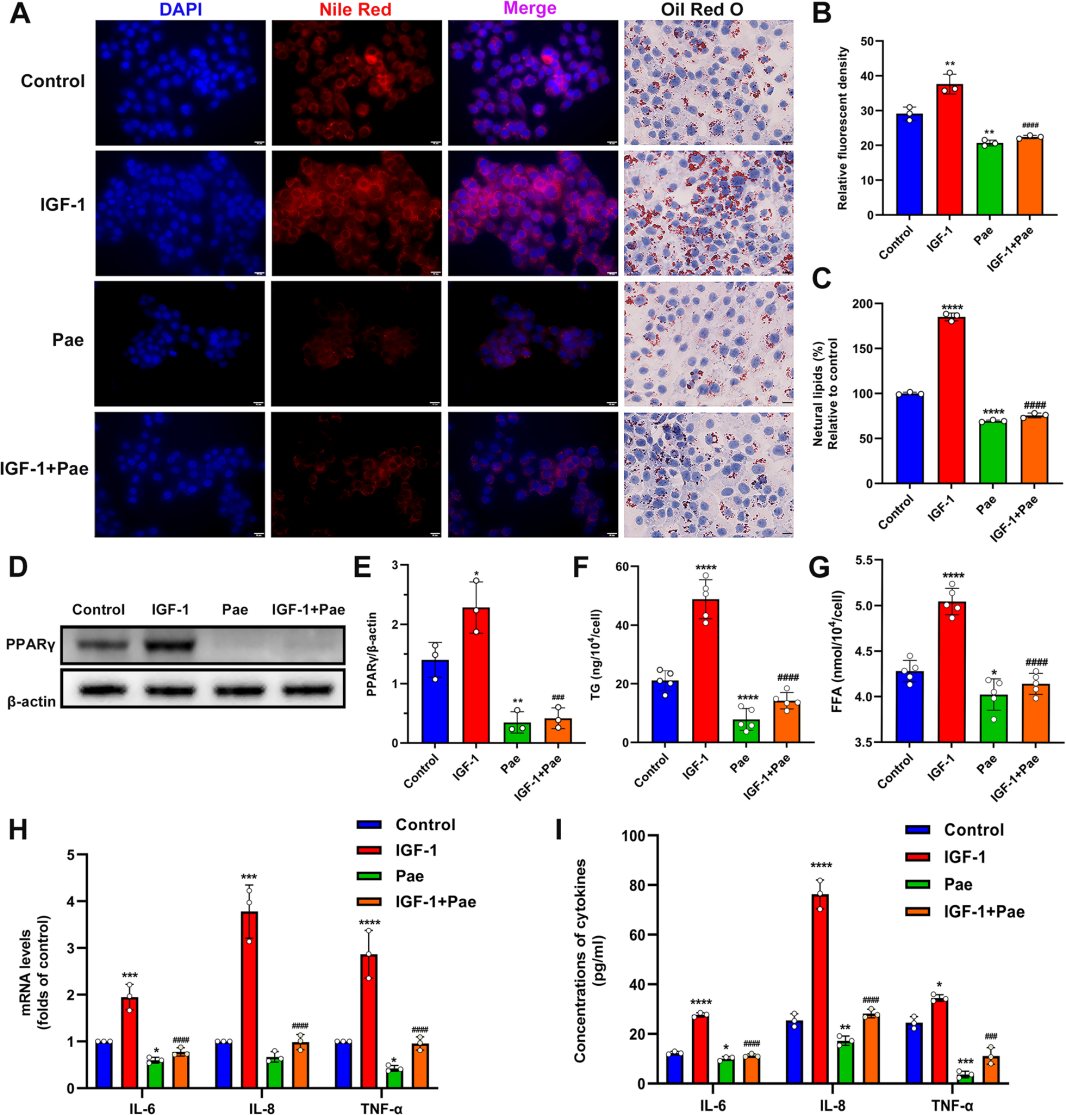
Effects of Pae on lipogenesis and inflammation in IGF-treated SZ95 sebocytes. After pre-treatment with 100 nM IGF-1 for 1 h, cells were then incubated with 80 µM Pae for 6 h. **A** Intracellular lipids were visualized by Nile Red and ORO staining. Scale bars = 20 μm. **B** Relative fluorescence of Nile red staining (*n* = 3). **C** The OD values of supernatant ORO levels (%) were measured at 500 nm. **D** Western blot detection of PPARγ, with β-actin as a loading control. **E** Densitometry analysis of the PPARγ protein (*n* = 3). **F** and** G** Spectrophotometric analysis of TG and FFA levels (*n* = 5). **H** qRT-PCR analysis for the transcript levels of IL-6, IL-8 and TNF-α (*n* = 3). **I** ELISA analysis of pro-inflammatory cytokines (IL-6, IL-8 and TNF-α) levels in cell culture supernatant (*n* = 3). Data were expressed as mean ± SD. *P < 0.05, **P < 0.01, ***P < 0.001, ****P < 0.0001 vs. untreated cells. ^###^*P* < 0.001, ^####^*P* < 0.0001 vs. IGF-1-treated cells

We next sought to investigate the potential impact of Pae on IGF-1-induced lipogenesis. The lipid droplets were stained with Oil Red O (ORO) and Nile Red dyes to detect neutral lipids. Our results revealed an augmented intracellular lipid accumulation in SZ95 sebocytes treated with IGF-1, which was partially inhibited by Pae treatment (Fig. 3A–C). To understand the molecular basis underlying sebo-suppression induced by Pae, we examined the expression of the lipogenic transcription factor PPARγ through Western blotting. The protein levels of PPARγ were upregulated in IGF-1-treated cells, whereas this induction was significantly attenuated upon Pae administration (Fig. 3D and E). To further investigate the effects of Pae on sebum composition, we measured the levels of main neutral lipids, including Triglyceride (TG) and Free Fatty Acids (FFA). The levels of TG and FFA were increased by IGF-1, while these lipids were significantly reduced when Pae was administered (Fig. 3F and G). These findings indicate that Pae has the potential to inhibit the acne-like sebaceous lipogenesis pattern induced by IGF-1.

Previous research demonstrated a positive correlation between decreased sebum production and improvement in the severity of acne lesions [22]. PPARγ was upregulated in SGs derived from acne patients compared to those from healthy individuals [23], and its agonists can stimulate sebocyte differentiation and increase the rate of sebum excretion [24]. PPARγ activation serves as an essential co-factor for the induction of sebum production by IGF-1 [25]. In HFD-induced obese mice, Pae alleviated lipid accumulation in liver and adipose tissue by downregulating PPARγ [26, 27]. Our investigation supported prior findings by demonstrating a general rise in PPARγ expression in IGF-1-treated SZ95 sebocytes, while the administration of Pae effectively reversed the effects.

The acne pathogenesis involves excessive sebum secretion and intricate changes in lipid homeostasis [28]. PPARγ activation alters the lipid composition, particularly affecting the biosynthesis of TG and FFA in SZ95 sebocytes [29]. On the skin surface, lipases catalyze the hydrolysis of TG into FFA. Recent findings have proved that the presence of saturated fatty acids in FFA can induce lipid accumulation in cultured sebocytes [30]. Moreover, FFA can stimulate the inflammatory response in human sebocytes [31]. As a potent anti-inflammatory agent, the relationship between Pae and FFA-induced sebaceous inflammation is still unknown and requires further research.

### Pae inhibited IGF-1-induced inflammation in SZ95 sebocytes

To evaluate the anti-inflammatory properties of Pae, the gene expression levels of L-6, IL-8, and TNF-α in SZ95 sebocytes were assessed using qRT-PCR after treatment with Pae. A significant reduction in the mRNA levels of IL-6, IL-8, and TNF-α was observed in Pae-treated SZ95 sebocytes compared to untreated cells. Furthermore, the increase in mRNA levels of IL-6, IL-8, and TNF-α caused by IGF-1 was significantly attenuated following treatment with Pae (Fig. 3H). Consistently, results from the ELISA analysis demonstrate that Pae was able to decrease the secretory protein levels of IL-6, IL-8, and TNF-α in IGF-1-treated sebocytes, further supporting the inhibitory effect of Pae on the inflammatory response activated by IGF-1. To further validate the findings, ELISA was performed to quantify the protein levels of IL-6, IL-8, and TNF-α. Consistently, the secretion of these cytokines was reduced in both normal and IGF-1-treated SZ95 sebocytes after exposure to Pae (Fig. 3I). These results indicate the inhibitory effect of Pae on the inflammatory response activated by IGF-1 in SZ95 sebocytes.

In addition to its lipogenic activities, IGF-1 plays a crucial role in the development of acne-associated perifollicular inflammation by upregulating the expression of pro-inflammatory cytokines such as IL-1β, IL-6, IL-8, and TNF-α [6]. The effect of Pae in disrupting LPS-stimulated renal inflammation has been previously demonstrated by reducing the release of IL-1β, IL-6 and TNF-α [32]. Our experiments used IGF-1 as a stimulator of cytokine synthesis, thereby inducing the transactivation of cytokine genes in acne pathogenesis and forming an in vitro model of sebaceous inflammation. In this study, we found that Pae abolished the secretion of IGF-1-induced pro-inflammatory factors from SZ95 sebocytes induced by IGF-1. Additionally, Pae downregulated the gene expression of IL-6, IL-8, and TNF-α, indicating its anti-inflammatory effect against IGF-1.

### Inhibition of PI3K augments the attenuation of lipogenesis in Pae-treated SZ95 sebocytes

**Fig. 4:**
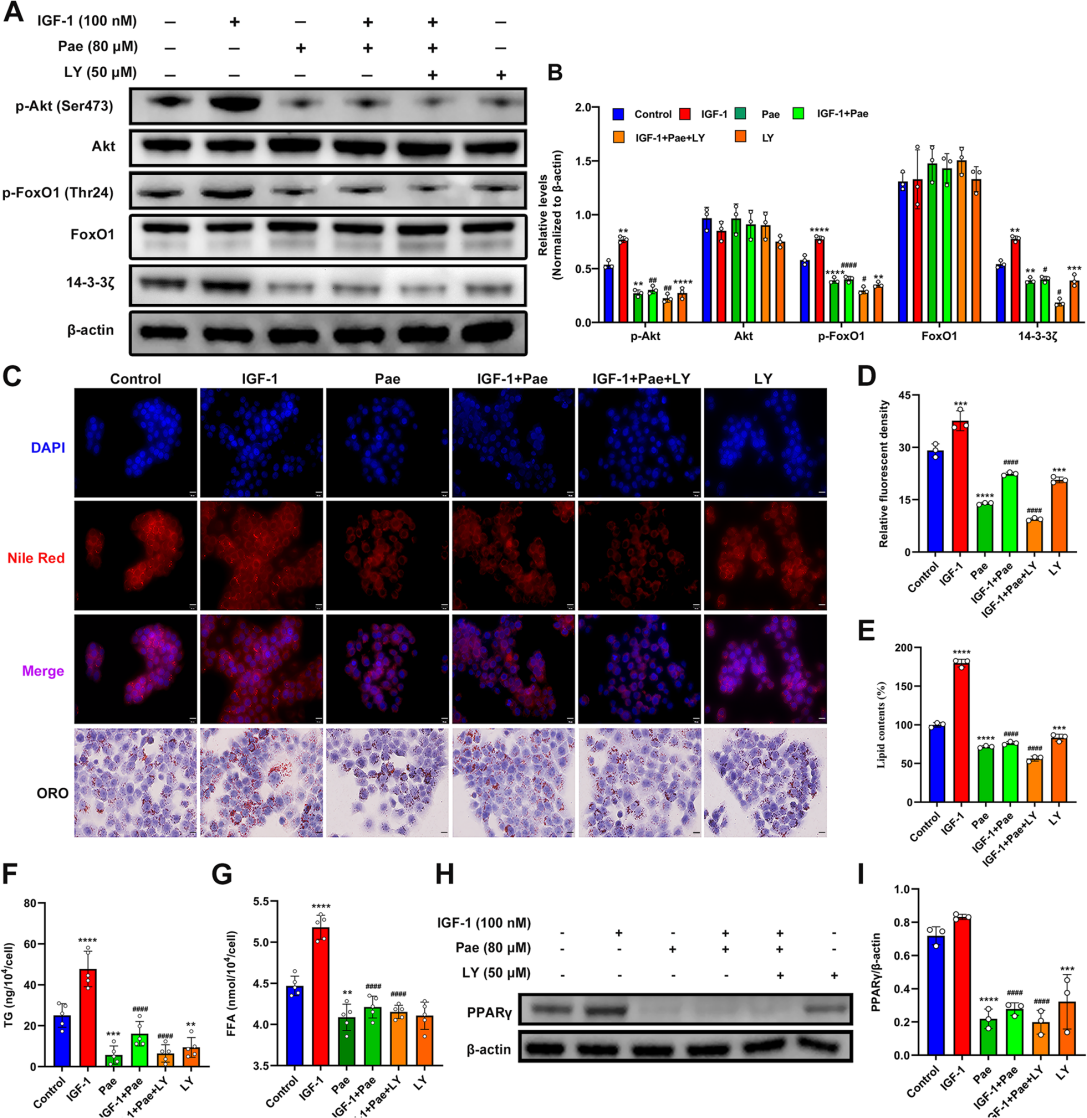
Pae mitigated IGF-1-induced lipogenesis in SZ95 sebocytes by targeting the PI3K/Akt/FoxO1 pathway. Following IGF-1 stimulation, cells were treated with 80 µM Pae alone for 6 h or in combination with 50 µM LY for 30 min. **A **and** B** Representative blots and quantitative analyses for p-Akt, Akt, p-FoxO1, FoxO1, and 14-3-3ζ expression in the untreated or IGF-1 and Pae-treated SZ95 sebocytes, with the protein intensities normalized to β-actin (*n* = 3). **C** Lipid droplets were visualized using Nile Red and ORO staining. Scale bars = 20 μm. **D** Relative fluorescence of Nile red staining (*n* = 3). **E** The OD values of supernatant ORO levels (%) were measured at 500 nm. **F **and** G** Quantitation of TG and FFA levels were spectrophotometrically measured (*n* = 5). **H** and **I** Representative blotting images and relevant quantitation of PPARγ normalized to β-actin (*n* = 3). Data were expressed as mean ± SD. **P < 0.01, ***P < 0.001, ****P < 0.0001 vs. untreated cells. ^#^*P* < 0.05, ^##^*P* < 0.01, ^####^*P* < 0.0001 vs. IGF-1-treated cells

To investigate whether the effects of Pae in combating IGF-1-induced lipogenesis were mediated by the PI3K/ Akt/FoxO1 pathway, we examined the effects of Pae under the administration of PI3K inhibitor LY294002 (LY) in SZ95 sebocytes. Protein expression of p-Akt and p-FoxO1 were significantly upregulated by IGF-1, but downregulated by Pae and LY compared to untreated cells (Fig. 4A and B). Notably, the inhibitory effect of Pae was enhanced by the co-intervention of LY. However, there was no effect on the total levels of Akt and FoxO1. In addition, it has been shown that 14-3-3 inactivates FoxO1 by promoting the nuclear exclusion of p-FoxO1 [33]. Our result showed that protein levels of 14-3-3ζ were markedly decreased by Pae or co-treatment with LY, indicating that Pae might prevent the degradation of FoxO1 through inhibiting the activity of 14-3-3ζ. These results suggest that Pae could function as an antagonist of IGF-1 by inhibiting the phosphorylation of downstream PI3K/Akt/FoxO1 pathway.

Lipids staining was used to validate the findings above. Exposure to Pae, either alone or co-treated with LY effectively mitigated lipid overproduction induced by IGF-1 (Fig. 4C–E). Moreover, the combined treatment of Pae and LY substantially reversed the IGF-1-stimulated synthesis of TG and FFA (Fig. 4F, G), and attenuated the protein expression of PPARγ induced by IGF-1 (Fig. 4H). Collectively, these data suggest that the inhibitory effects of Pae on IGF-1-induced lipogenesis are, at least partially, attributed to the inactivation of the PI3K/Akt/FoxO1 pathway.

The transcriptional activity of FoxO1 relies on a nuclear-cytoplasmic transport system. IGF-1 triggers Akt-mediated FoxO1 phosphorylation through the PI3K signaling pathway, which ultimately results in FoxO1 degradation [7]. Studies have shown that Pae inhibited the PI3K/Akt pathway and restore FoxO1 expression, which may contribute to its anti-cancer effects [34, 35]. In our study, we found that Pae treatment significantly diminished the phosphorylation of Akt and FoxO1 induced by IGF-1. By binding to 14-3-3, p-FoxO1 undergoes conformational changes and travels from the nucleus to the cytoplasm for degradation [33]. Our results displayed that Pae decreased 14-3-3ζ expression in IGF-1-stimulated SZ95 sebocyte, suggesting that Pae can indirectly improve the nuclear retention of FoxO1. Nuclear retention of FoxO1 restrains the transcriptional activity of the PPAR family [36], which may explain the downregulation of PPARγ after Pae treatment. These findings imply that Pae may function as an inhibitor of the PI3K/Akt/FoxO1 pathway, thereby normalizing IGF-1-induced dysregulated lipogenesis. Moreover, we found an increase in TG and FFA production in response to IGF-1, while the overproduction of TG and FFA induced by IGF-1 was counteracted following LY incubation, suggesting that IGF-1 may disturb sebum composition through the PI3K pathway. Pae normalized sebaceous lipidomics by reducing the production of TG and FFA in IGF-1-treated SZ95 sebocytes through inhibition of the PI3K pathway, which may underpin the therapeutic effectiveness of Pae in acne therapy.

### JAK2 inhibition blocked the anti-inflammatory effects of Pae in IGF-1-treated SZ95 sebocytes

**Fig. 5:**
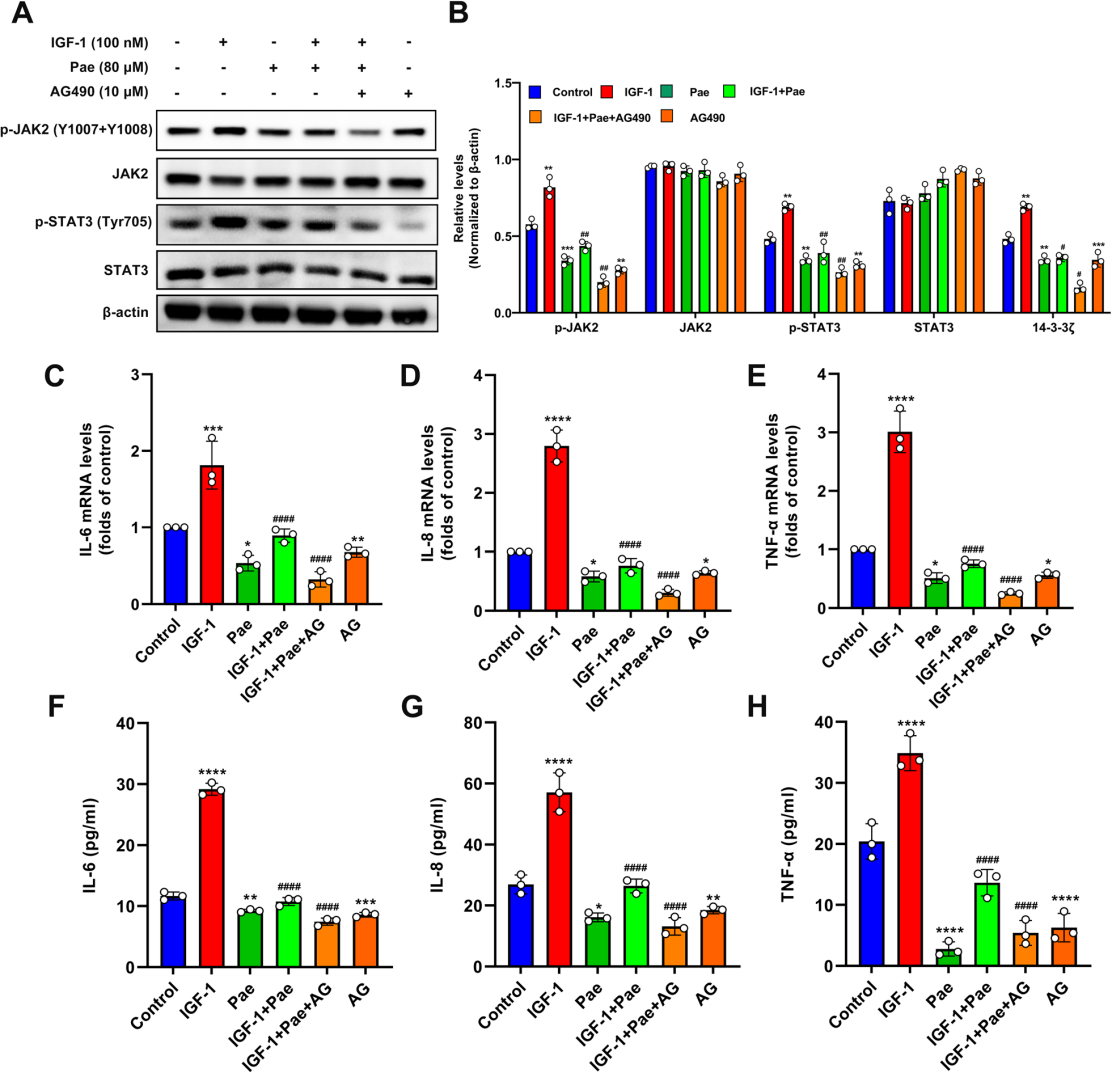
Pae alleviated IGF-1-induced inflammation in SZ95 sebocytes by inhibiting the JAK2/STAT3 pathway. Upon stimulation with IGF-1, cells were treated with 80 µM Pae for 6 h, or co-treated with 10 µM of AG for 2 h. **A **and** B** Representative blots for p-JAK2, JAK2, p-STAT3, and STAT3 are shown, along with quantification of protein intensities normalized to β-actin. **C**–**E** The mRNA levels of IL-6, IL-8 and TNF-α were evaluated using qRT-PCR. **F**–**H** Quantitative analyses of supernatant IL-6, IL-8 and TNF-α were measured using ELISA. Data were expressed as mean ± SD, *n* = 3. *P < 0.05, **P < 0.01, ***P < 0.001, ****P < 0.0001 vs. untreated cells. ^#^*P* < 0.01, ^##^*P* < 0.001, ^####^*P* < 0.0001 vs. IGF-1-treated cells

Previous reports have implicated the protective effects of Pae on inflammatory progression by blocking the JAK2/STAT3 pathway in immune cells [12]. To explore whether Pae inhibited IGF-1-induced inflammation by modulating the JAK2/STAT3 pathway, we analyzed the impact of Pae under the intervention of the JAK2 inhibitor AG490 (AG) in SZ95 sebocytes. Our results indicated that Pae significantly decreased the expression of p-JAK2 and p-STAT3 induced by IGF-1 without effect on total levels of JAK2 and STAT3. The dephosphorylation of JAK2 and STAT3 induced by Pae was remarkably augmented in the presence of AG (Fig. 5A and B). To confirm our expectations, the levels of pro-inflammatory cytokines were analyzed by qRT-PCR. It was shown that co-incubation of Pae and AG significantly attenuated the mRNA levels of the IL-6, IL-8 and TNF-α in response to IGF-1 stimulus compared to the cells treated with IGF-1 and Pae (Fig. 5C–E). Consistent with this, the secreted IL-6, IL-8 and TNF-α protein levels were also decreased following Pae and AG treatment, as detected by ELISA (Fig. 5F–H). Overall, these findings suggest that targeting JAK2 can potentiate the anti-inflammatory effects of Pae.

It has been verified that Pae protected against oxidative stress and alleviated TNF-α and IL-6 expression induced by oxygen-glucose deprivation in PC12 cells by modulating the JAK2/STAT3 pathway [37]. In SZ95 sebocytes, STAT3 activation by leptin contributes to the increased expression of proinflammatory cytokines including IL-6, IL-8, TNF-α and IL-1β, thereby promoting inflammation [10]. Consistent with previous studies, our results showed a decrease in the phosphorylated levels of JAK2 and STAT3 in Pae-treated SZ95 sebocytes, while JAK2 inhibition enhanced the inhibitory effect of Pae on attenuating the production of IL-6, IL-8 and TNF-α. Alternatively, STAT3 activation could result in an anti-inflammatory phenotype by increasing the expression of anti-inflammatory cytokine such as IL-10 in immune cells [38]. Previous studies reported that both the activation of IGF-1 receptor and IGF-1 exposure promote the binding and phosphorylation of JAK2 and STAT3 protein, thereby enhancing the immunosuppressive activity of murine T cell types [11]. In contrast, our analysis found that abolishment of the JAK2/STAT3 pathway or co-treatment with Pae attenuated the production of IL-6, IL-8 and TNF-α induced by IGF-1, suggesting that IGF-1 may stimulate inflammation in SZ95 sebocytes through activation of the JAK2/STAT3 pathway. Based on these findings, the roles of IGF-1 and JAK2/STAT3 in inflammatory processes can vary depending on the specific cell types and species involved. Further research is required to elucidate whether IGF-1 induces pro-inflammatory responses through the activation of JAK2/STAT3 pathway using in vitro models for acne.

## Conclusion

In conclusion, our study demonstrated the multifaceted impacts of Pae on SZ95 sebocytes, including its roles in proliferation, cell cycle dynamics, apoptosis, lipogenesis, and inflammatory processes. Furthermore, we have also uncovered that Pae exerted antergic effects on IGF-1-induced lipogenesis and inflammation, potentially through the inactivation of the PI3K/Akt/FoxO1 and JAK2/STAT3 signaling pathways. However, the potential of Pae as a viable pharmacological approach for the treatment of acne requires further exploration through animal experiments or clinical trials.

## Experimental section

### Cell line, cultivation, and treatments

SZ95 sebocytes, a immortalized human sebaceous gland cell line, were utilized in this study with the approval of Professor Christos C. Zouboulis [39]. Cells were cultivated in Sebomed basal medium (Sigma-Aldrich, St. Louis, MI, USA) supplemented with 10% fetal bovine serum (Gibco, Rockville, MD), 5 ng/ml recombinant human epidermal growth factor (Invitrogen, CA) and 1% penicillin/streptomycin in a humidified incubator containing 5% CO_2_ at 37 °C. Pae (purity: >98%) was purchased from GLPBIO (Montclair, CA, USA). IGF-1 (purity: ≥98%), PI3K inhibitor LY294002 (purity = 99.97%) and JAK2 inhibitor AG490 (purity = 99.66%) were purchased from APExBIO Technology LLC (Houston, USA). All of these pharmaceuticals were solubilized in dimethyl sulfoxide (Sigma-Aldrich, USA). To eliminate the impact of serum, cells were initially cultured in a serum-free medium for 24 h prior to pharmaceutical interventions. To induce an in vitro microenvironment prone to acne, cells were pretreated with 100 nM IGF-1 for 1 h.

### Cell viability assay in SZ95 sebocytes

Cells were cultivated in 96-well plate at a density of 5,000 cells per well for 24 h. After the indicated treatments, cells were incubated in the fresh medium with 10 µl CCK-8 solution for 40 min. The optical density (OD) value of supernatant formazan was measured at 450 nm using a VICTOR Nivo™ System (PerkinElmer, Waltham, USA).

### Crystal violet assay

Cells were grown on 6-well plates at a density of 10,000 per well overnight. After indicated treatments, the culture medium was removed, and cells were incubated in fresh complete medium containing 10% serum for an additional 24 h. After fixation with 4% formaldehyde for 30 min, cells were washed with PBS twice and stained with 500 µl crystal violet staining solution for 10 min at room temperature (RT). Stained cells were under air-drying for 10 min and then visualized by microscopy. Crystal violet was extracted and quantified according to a previously described method [20].

### Flow cytometry analysis

Cells were collected by centrifugation at 500×*g* for 5 min and resuspended in binding buffer. For cell cycle detection, cells were fixed with 70% ethanol overnight at 4 °C, resuspended with 500 µl staining buffer, and incubated with 10 µl mixture of PI and RNase A for 30 min. For apoptosis detection, approximately 1 × 10^5^ cells were incubated with 5 µl Annexin V-FITC and 5 µl propidium iodide (PI) for 15 min in the dark. After incubation, cells were gently vortexed and then analyzed using CytoFLEX Flow Cytometer (Beckman Coulter, USA). The percentages of normal cells (Annexin V-FITC^−^/PI^−^), cellular debris (Annexin V-FITC^−^/PI^+^), early apoptotic cells (Annexin V-FITC^+^/ PI^−^) and late apoptotic cells (Annexin V-FITC^+^/PI^+^) were calculated by FlowJo.10.8.1 software.

### AO/EB staining

The morphology of apoptotic cells was assessed using the AO/EB staining kit. Cells were centrifuged at 500×*g* for 5 min and then resuspend in Dilution Buffer at a concentration of 1 × 10^6^ cells/ml. Each 25 µl of the cell suspension was incubated with 1 µl AO/EB working solution for 5 min in the dark. After thorough mixing, images were captured using an inverted fluorescence microscope (Olympus Corporation, Tokyo, Japan).

### Lipid detection in SZ95 sebocytes

Cells were fixed with 4% paraformaldehyde at RT for 30 min and kept in dark place with 0.05% ORO or Nile red (10 µg/ml) staining solution for 30 min. After PBS washing, ORO-stained cells were counterstained with hematoxylin, and Nile red-stained cells were counterstained with DAPI. Intracellular lipids were visualized by fluorescence microscopy. The quantification of ORO levels was performed by measuring the absorbance of the supernatant following our previously reported protocol [40]. The fluorescent density of Nile red was semiquantitatively determined by using ImageJ software.

### Determination of TG and FFA

Approximately 5 × 10^6^ cells were harvested and lysed using lipid extraction buffer. After ultrasonication for 10 min, the supernatant was obtained and transferred to tubes for further purification through centrifugation at 10,000 ×*g* for 10 min. Following the manual instructions, the levels of TG and FFA were measured at 510 nm and 715 nm, respectively, using a microplate reader.

### Western blotting analysis

Cells was lysed in PRO-PREP protein extraction solution (Intron, Daejeon, Korea), and their concentrations was estimated by BCA. 20 µg per lane was separated by SDS-PAGE gel electrophoresis and then transferred onto PVDF membranes. Membranes were saturated in 5% non-fat milk (Coolaber, China) for 1 h at RT. After washing with Tris-buffered saline solution with Tween^®^20 (TBST) (Sigma-Aldrich, USA), the membranes were incubated overnight at 4 °C with diluted primary antibodies including p-Akt, Akt, p-FoxO1, FoxO1, p-JAK2, JAK2, p-STAT3, STAT3, 14-3-3ζ, PPARγ, and β-actin (1:1000). After washing with TBST buffer, the membranes were incubated with secondary antibodies (1:5000) and visualized by chemiluminescence. The mean density of each band was quantified using ImageJ software.

### Gene expression analysis

Total RNA was extracted using TRIzol Reagent (Life Technologies). 10 µg of total RNA were transcribed into first-strand cDNA through reverse transcription with M-MLV Reverse Transcriptase (Invitrogen). Aliquotes of cDNA mixture were amplified by polymerase chain reaction and analyzed using a Real-Time PCR Detection System with SYBR^®^ premix EX TaqTM (Takara Bio). PCR conditions were as follows: annealing temperature 60 °C, 30 cycles. The alterations in gene expression were described as fold increases relative to GAPDH. The primer sequences for genes are provided in Table 1.

**Table 1:** Primer sequences for qRT-PCR

Gene	Forward sequence (5′−3′)	Reverse sequence (5′−3′)
IL-6	ACTCACCTCTTCAGAACGAATTG	CCATCTTTGGAAGGTTCAGGTTG
IL-8	TTTTGCCAAGGAGTGCTAAAGA	AACCCTCTGCACCCAGTTTTC
TNF-α	CCTCTCTCTAATCAGCCCTCTG	GAGGACCTGGGAGTAGATGAG
GADPH	CAGCCTCAAGATCATCAGCA	TGTGGTCATGAGTCCTTCCA

### Enzyme-linked immunosorbent assay (ELISA)

The culture medium was collected and centrifuged at 1000×*g* for 10 min at 4 °C. The levels of IL-6, IL-8 and TNF-α was detected using ELISA assays (Solarbio, Beijing, China) according to the manufacturer’s protocols. The inflammatory cytokines in the sample were analyzed using a microplate reader, and their concentrations was quantified based on the established standard curve.

### Statistical analysis

Data are expressed as mean ± standard deviation (SD). Comparisons between two groups and multiple groups were analyzed by Student’s t-test and one-way analysis of variance (One-way ANOVA), respectively. Statistical significance was set at *p* < *0.05*.

## Supplementary Information

Supplementary material 1. 

## Data Availability

All data generated or analyzed during this study are included in this published article.
