# Supplementary material for: Paeoniflorin mitigates insulin-like growth factor 1-induced lipogenesis and inflammation in human sebocytes by inhibiting the PI3K/Akt/FoxO1 and JAK2/STAT3 signaling pathways
Source: Nat Prod Bioprospect. 2024 Oct 1;14(1):56. doi: 10.1007/s13659-024-00478-4 (PMC11442718; doi:10.1007/s13659-024-00478-4)

Supplementary Information

Paeoniflorin mitigates insulin-like growth factor 1-induced lipogenesis and inflammation in human sebocytes by inhibiting the PI3K/Akt/FoxO1 and JAK2/STAT3 signaling pathways

Chuanchuan Cai [a#], Si Liu [b, c#], Yufeng Liu [b, c#], Shaobin Huang [b, c], Shiya Lu[d], Fang Liu [b, c], Xiaohua Luo [b, c], Christos C. Zouboulis[e], Ge Shi [b, c*]

a. Department of Dermatology, Affiliated Hospital of Guangdong Medical University, Zhanjiang 524001, China.
b. Department of Cosmetic and Plastic Surgery, the Sixth Affiliated Hospital, Sun Yat-sen University, Guangzhou 510655, China.
c. Biomedical Innovation Center, the Sixth Affiliated Hospital, Sun Yat-sen University, Guangzhou 510655, China.
d. HUAMEI-BOND INTERNATIONAL COLLEGE, Guangzhou 510520, China.
e. Departments of Dermatology, Venereology, Allergology and Immunology, Staedtisches Klinikum Dessau, Brandenburg Medical School Theodor Fontane and Faculty of Health Sciences Brandenburg, Dessau 06847, Germany.

Supplementary Figure S1. Microscopic images for Fig. 1C. **(A-G)** Observation of cellular morphology of SZ95 sebocytes exposed to various doses of Pae. Original magnification, ×200.

(A) Pae (0 μM)

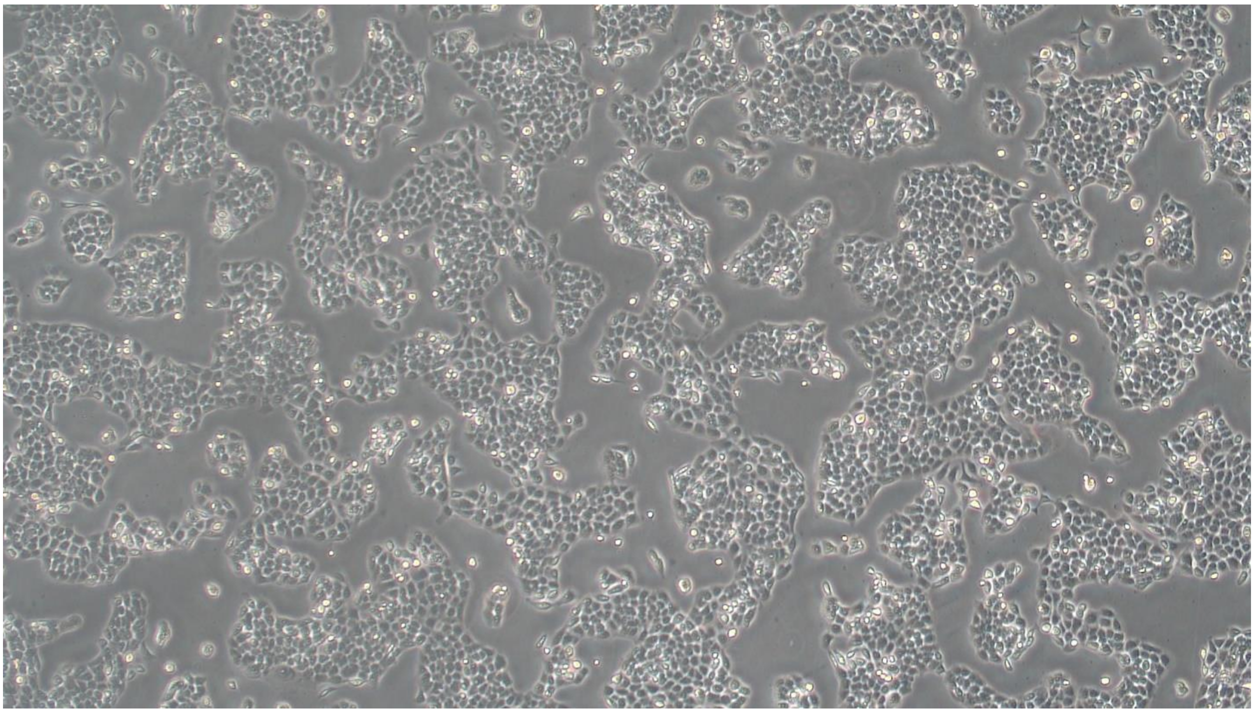

(B) Pae (10 μM)

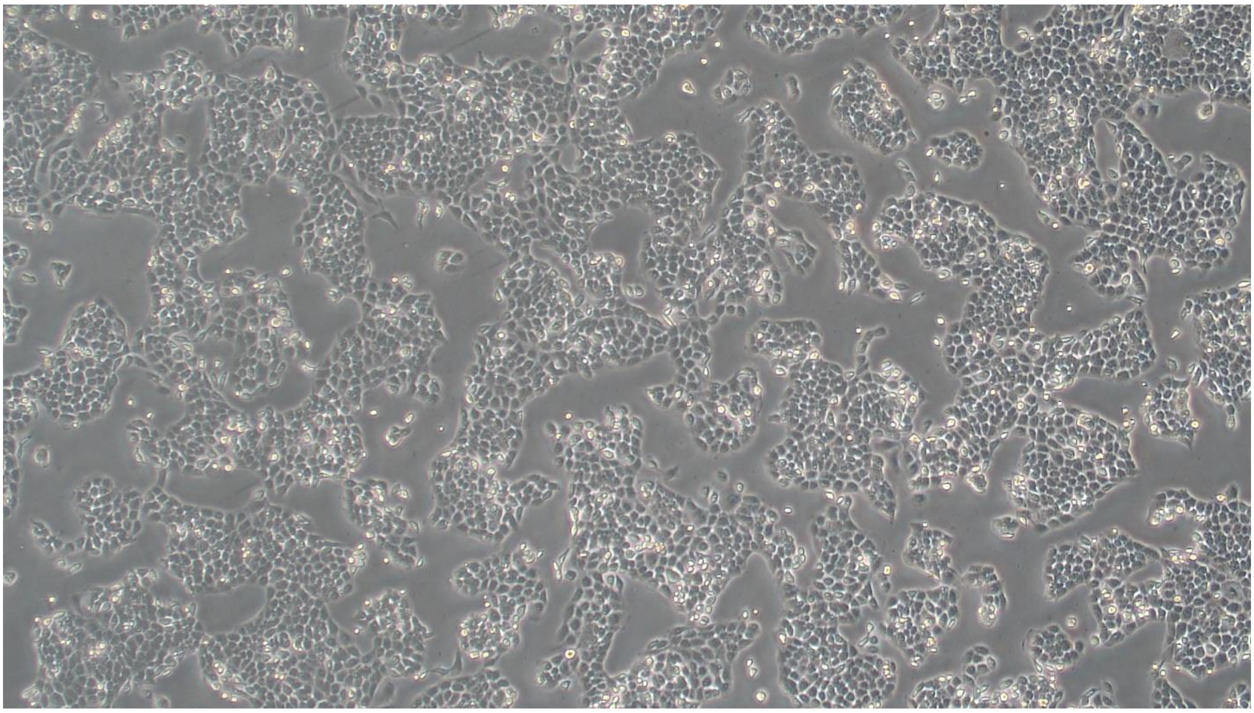

(C) Pae (20 μM)

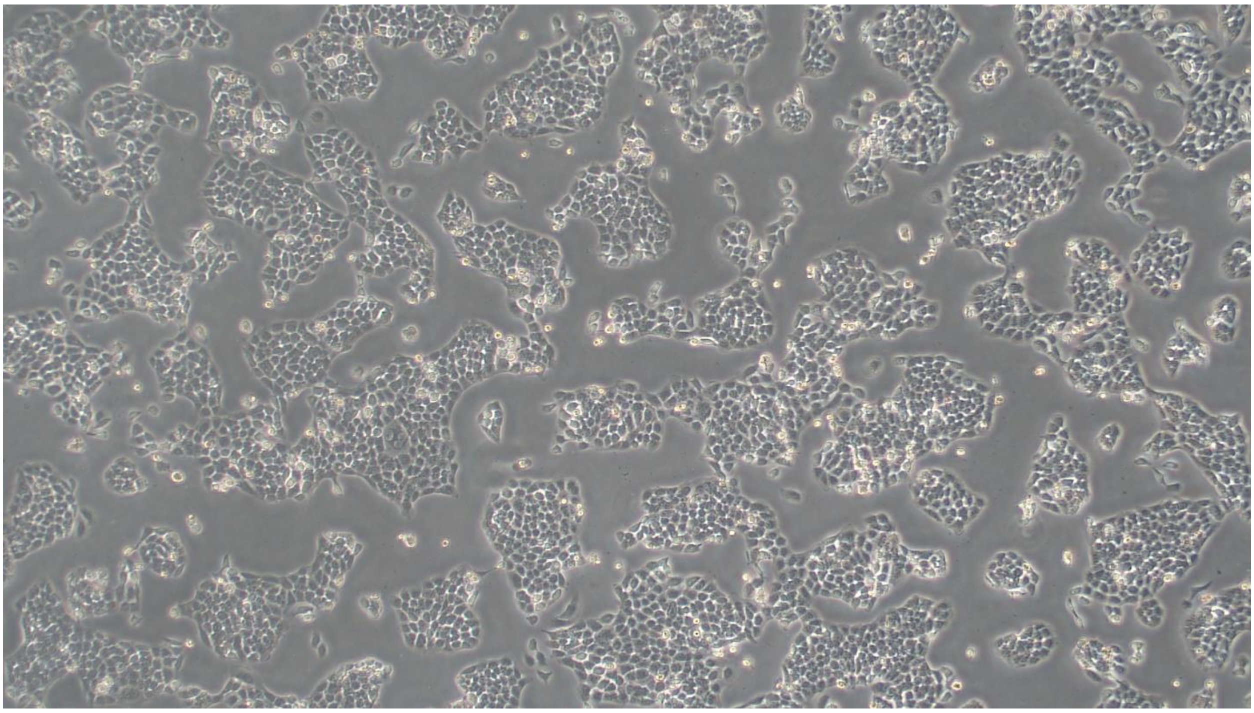

(D) Pae (40 μM)

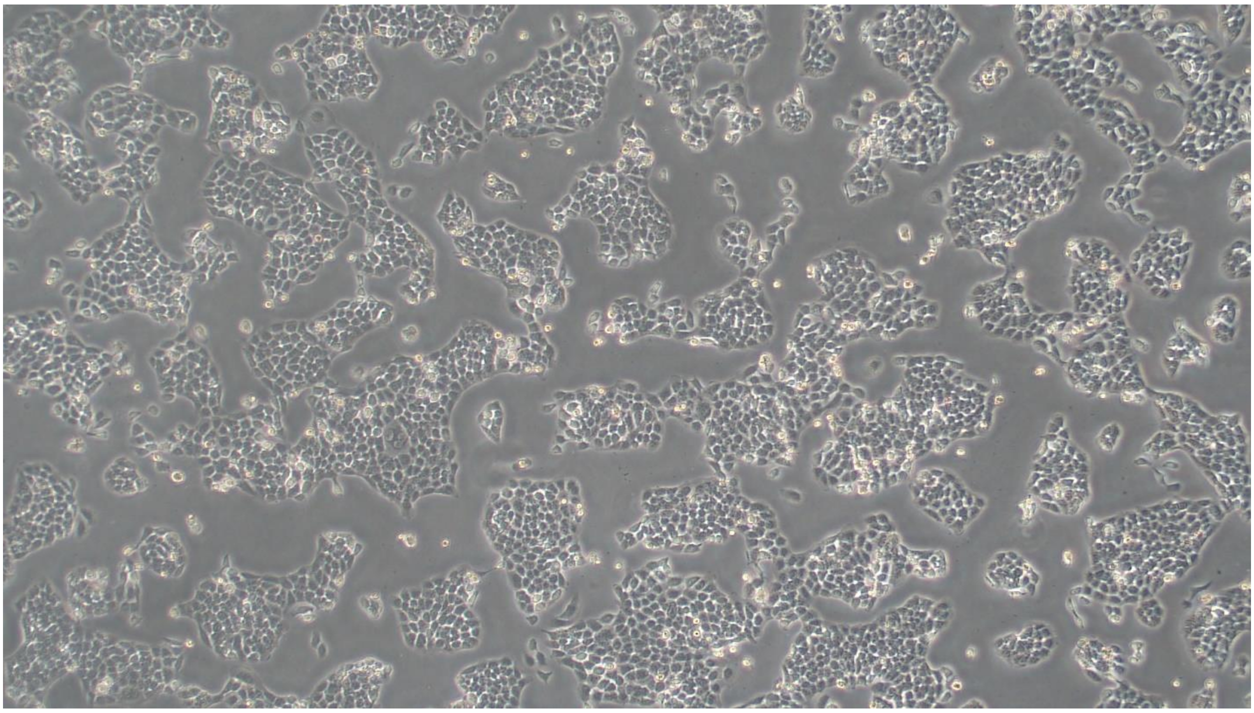

(E) Pae (80 µM)

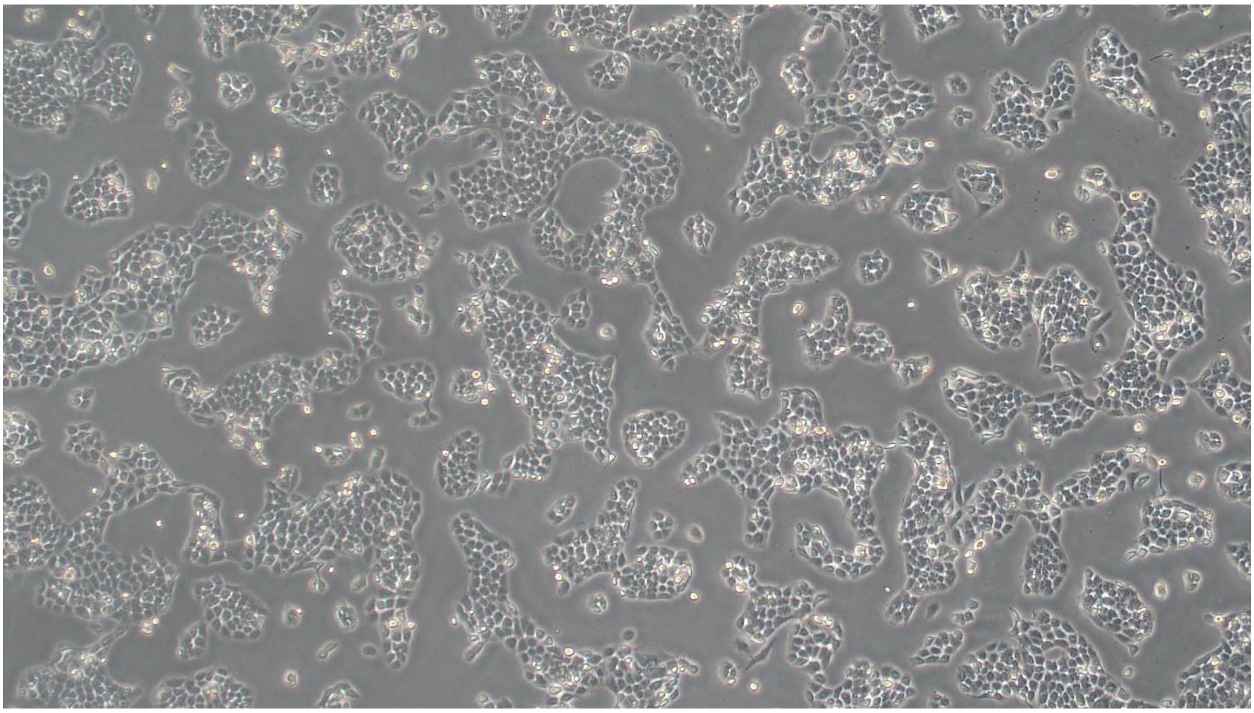

(F) Pae (160 µM)

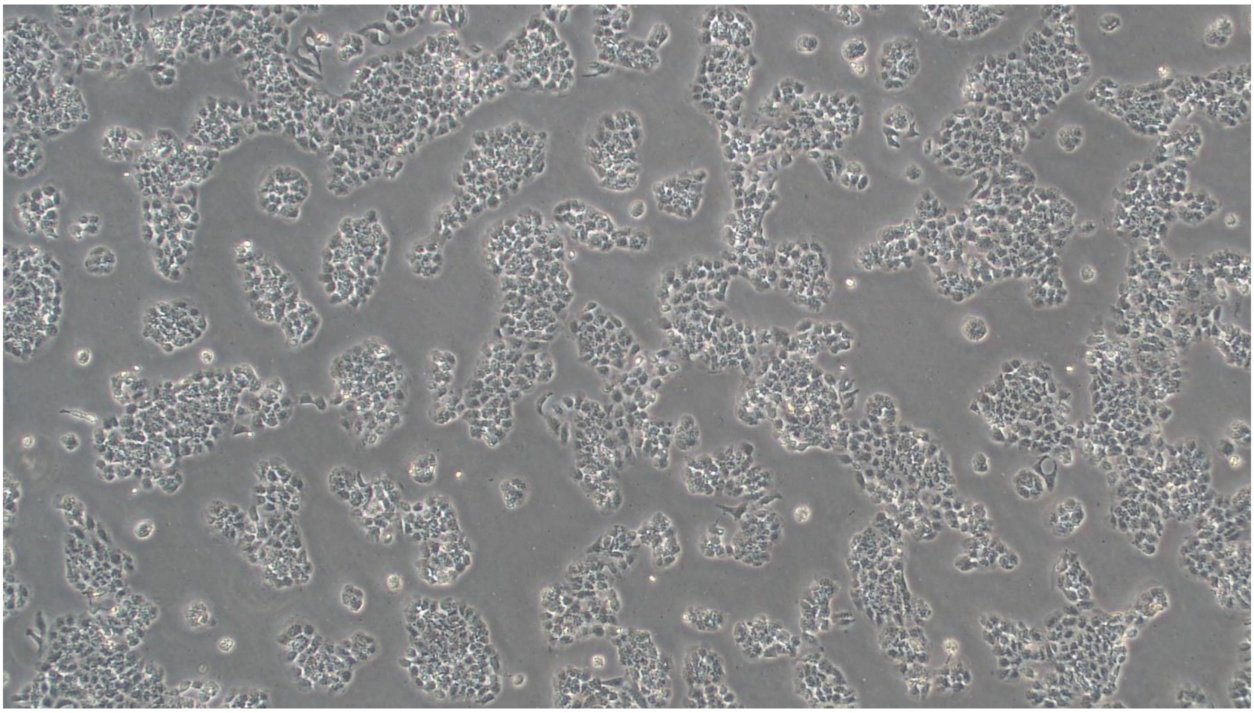

(G) Pae (200 μM)

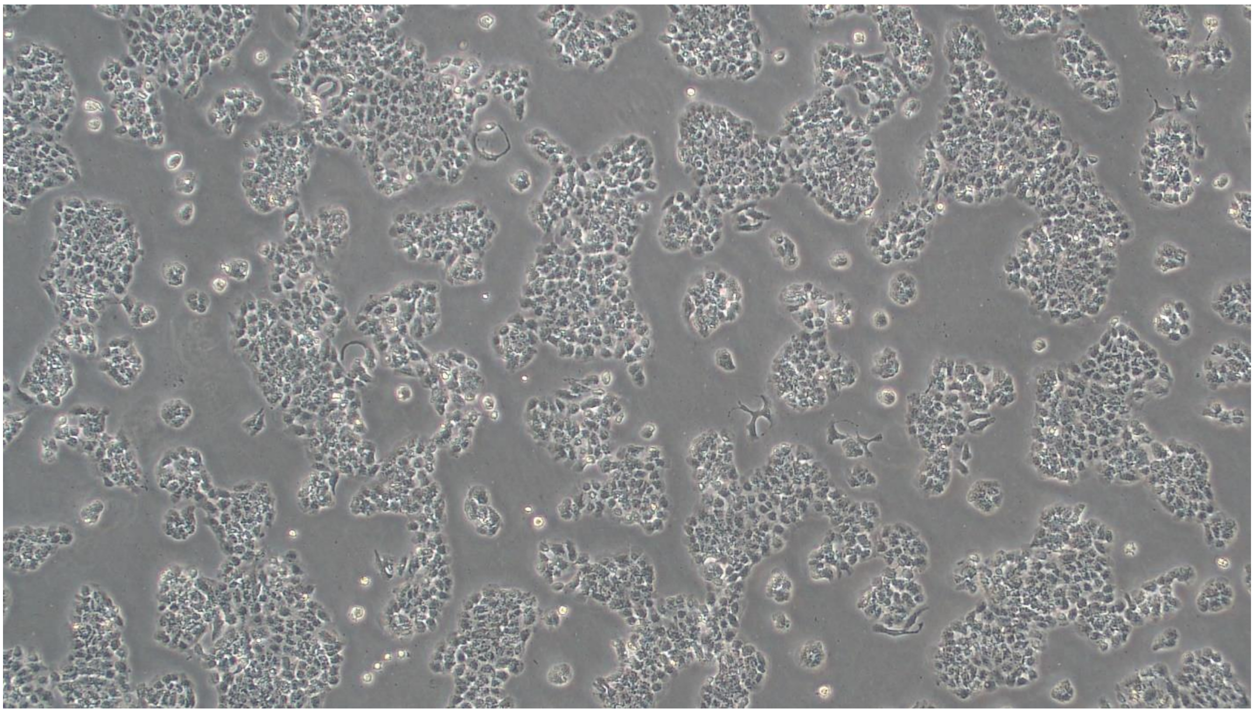

Supplementary Figure S2. Microscopic images for Fig. 2A. **(A-D)** Crystal violet staining for colony formation detection. Scale bar = 200 μm.

(A) Control group

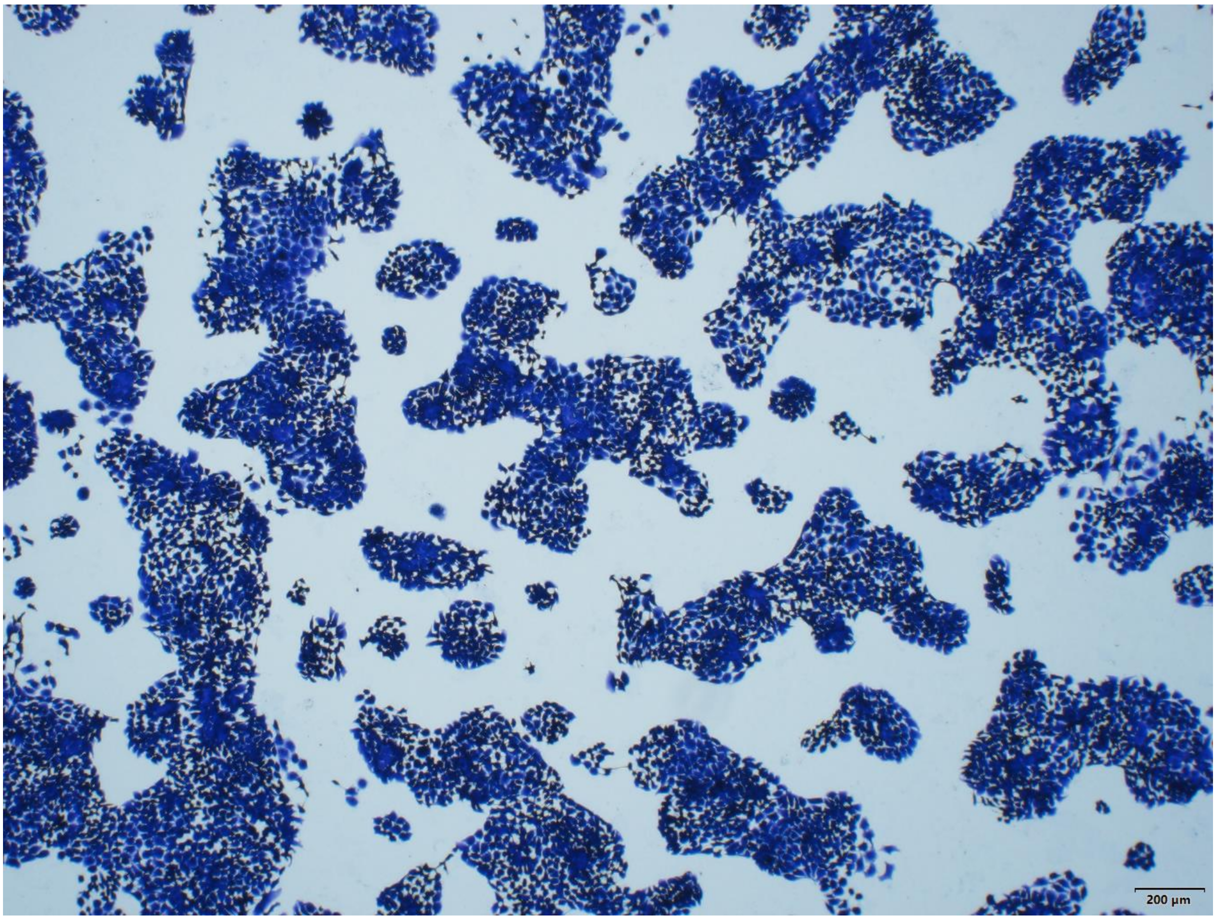

(B) IGF-1 group

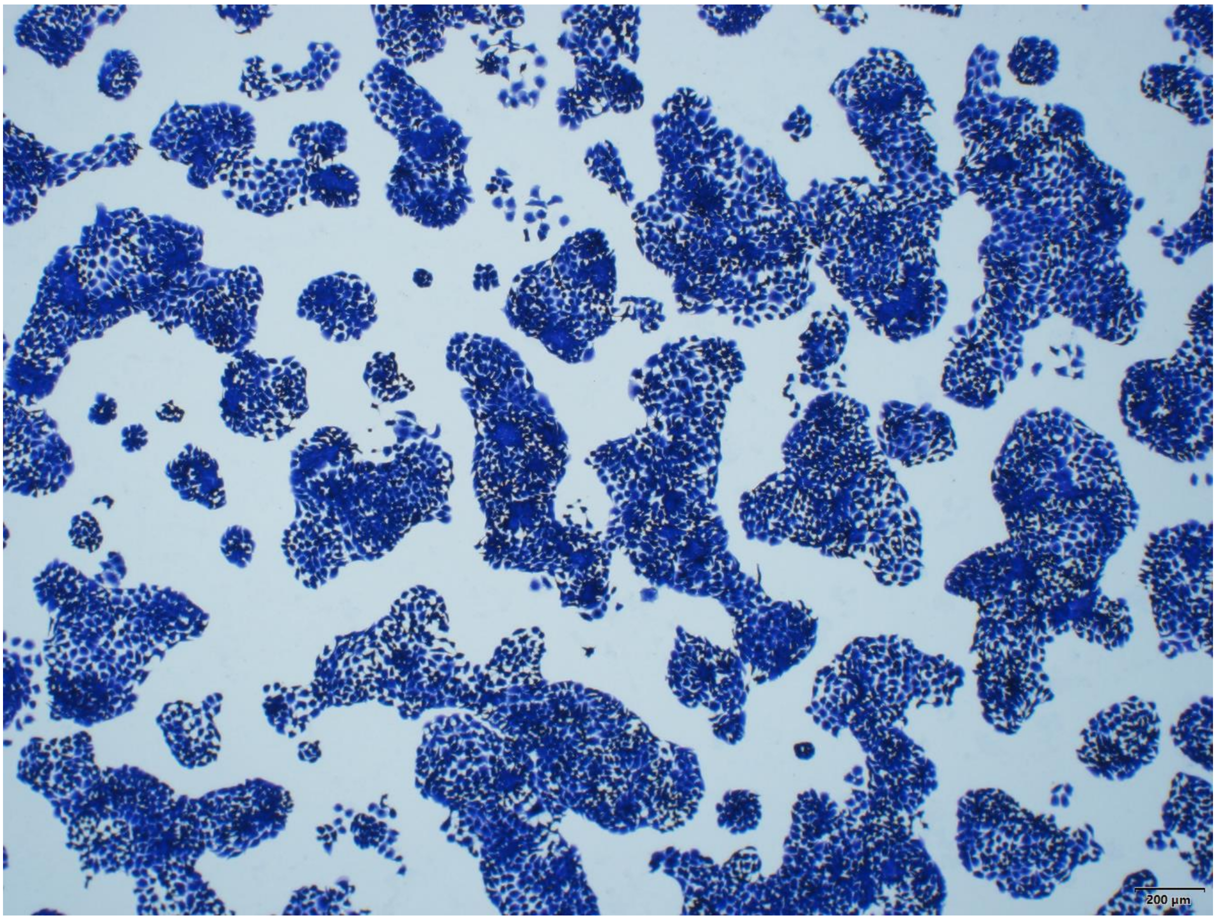

(C) Pae group

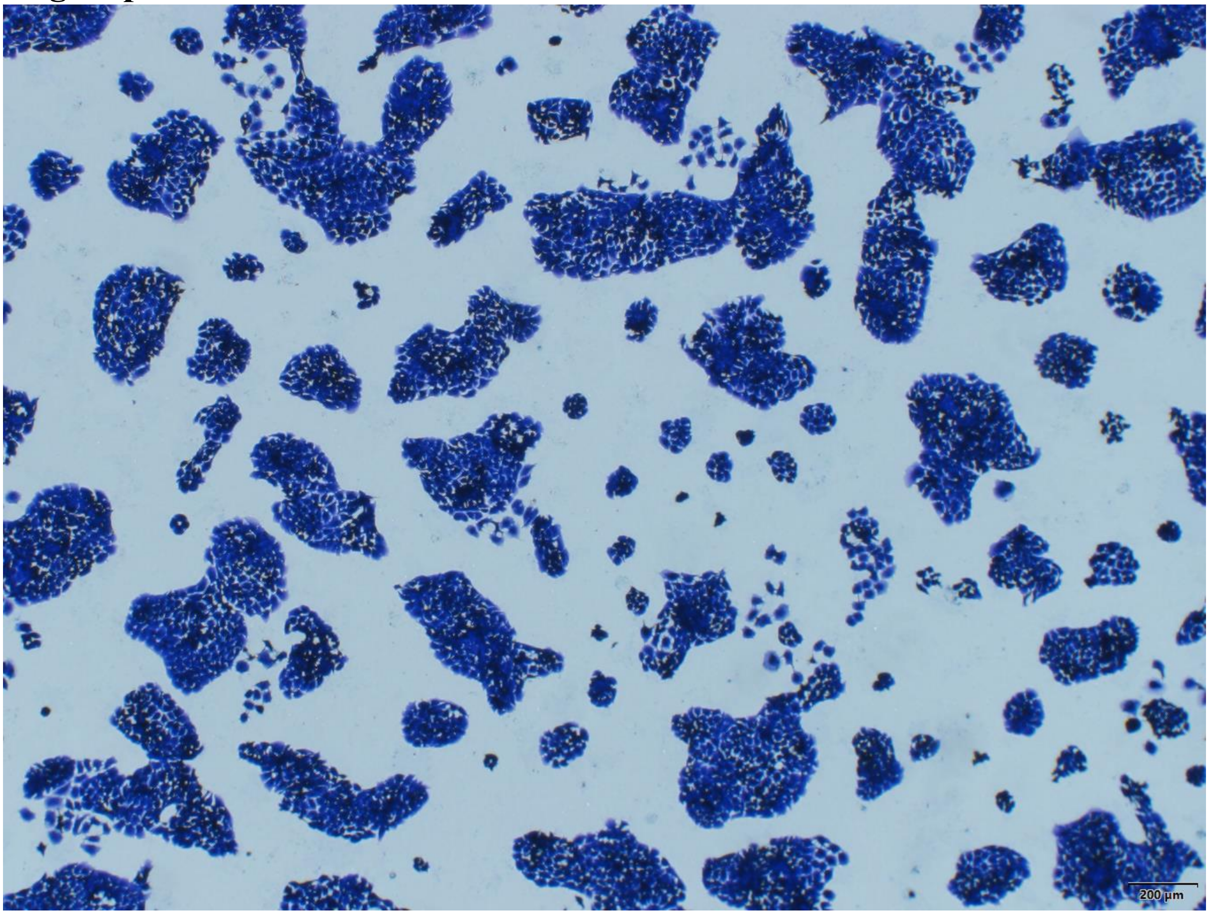

(D) IGF-1+Pae

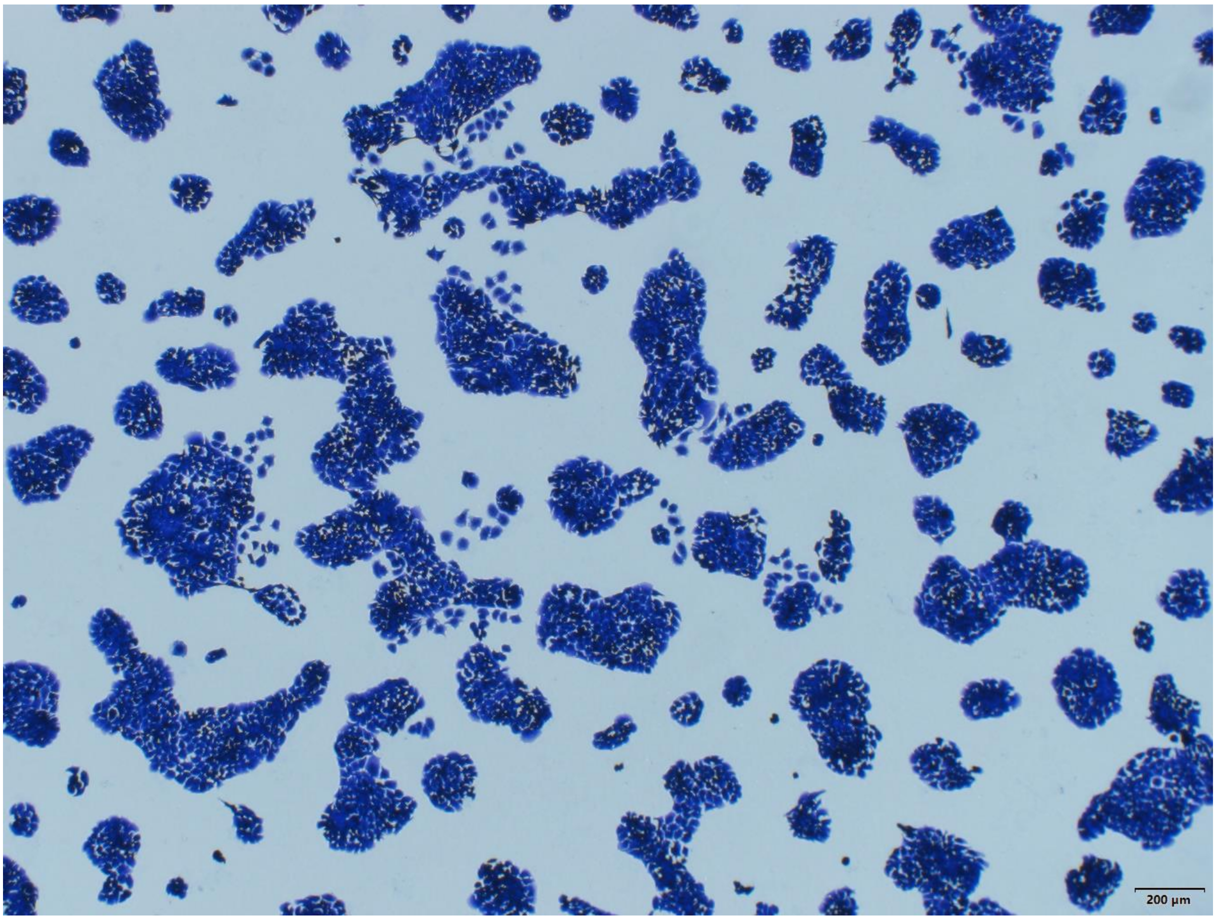

Supplementary Figure S3. Microscopic images for Fig. 2E. **(A-D)** Uncropped images for Fig. 2E. Scale bar = 100 μM.

(A) Control group

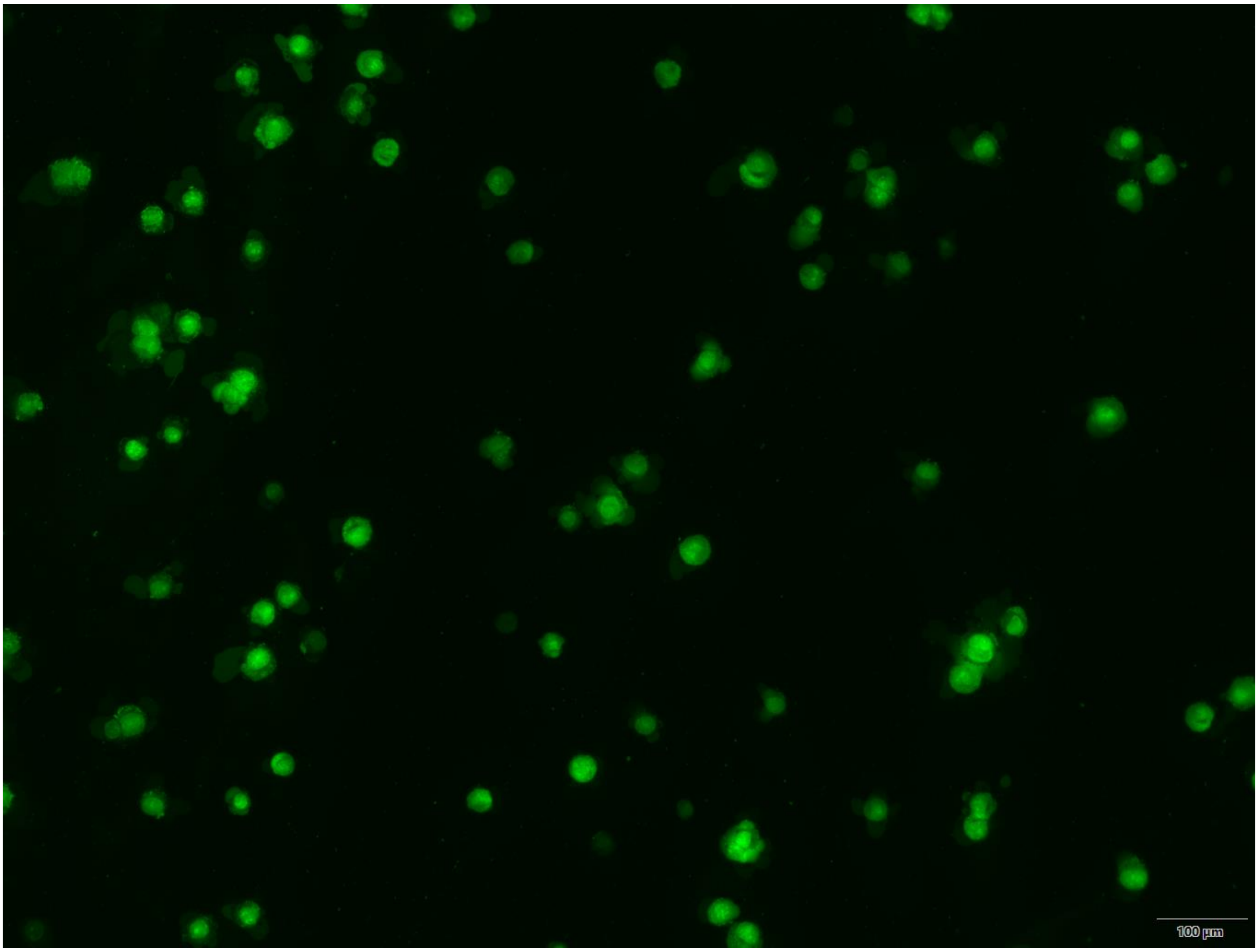

AO staining

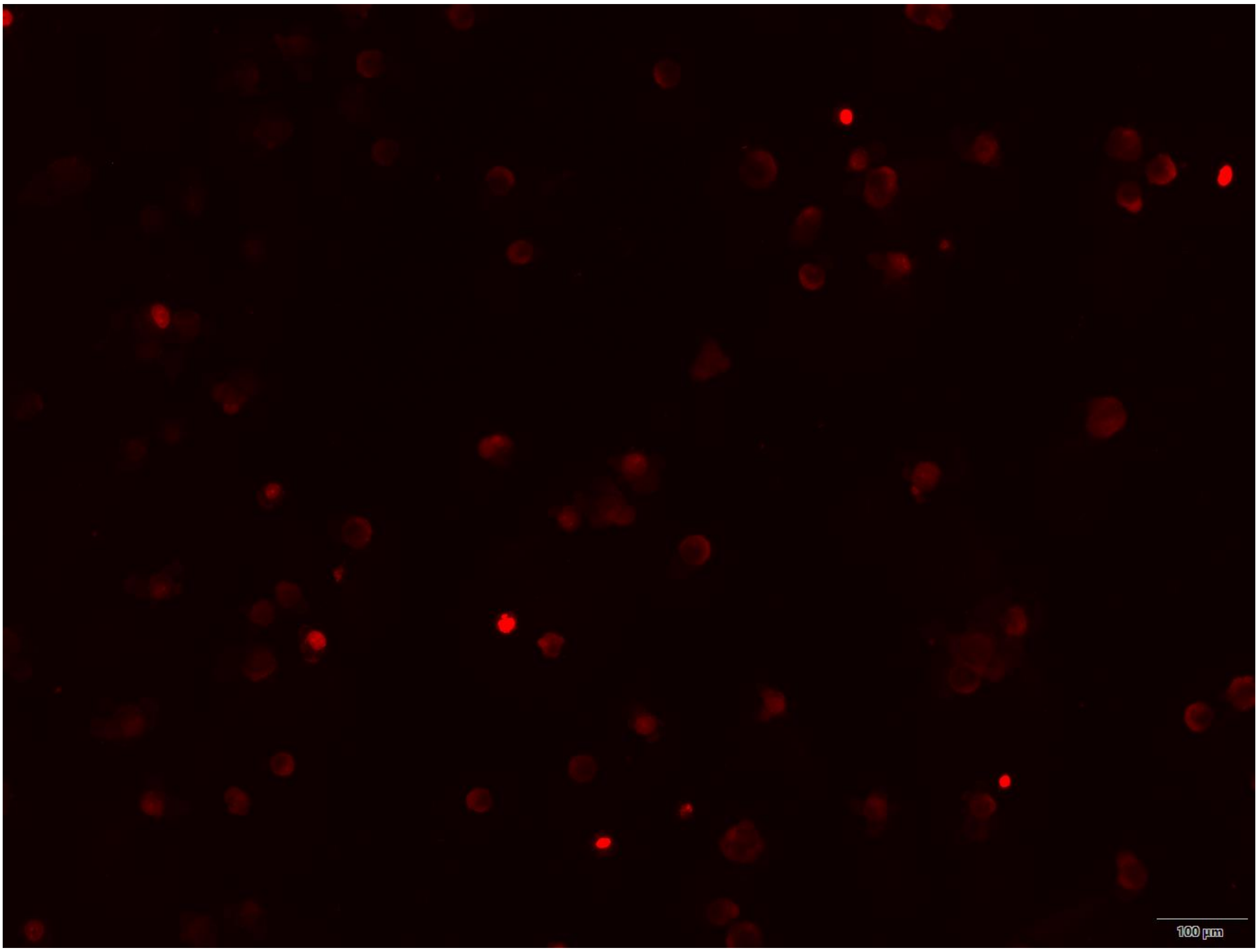

EB staining

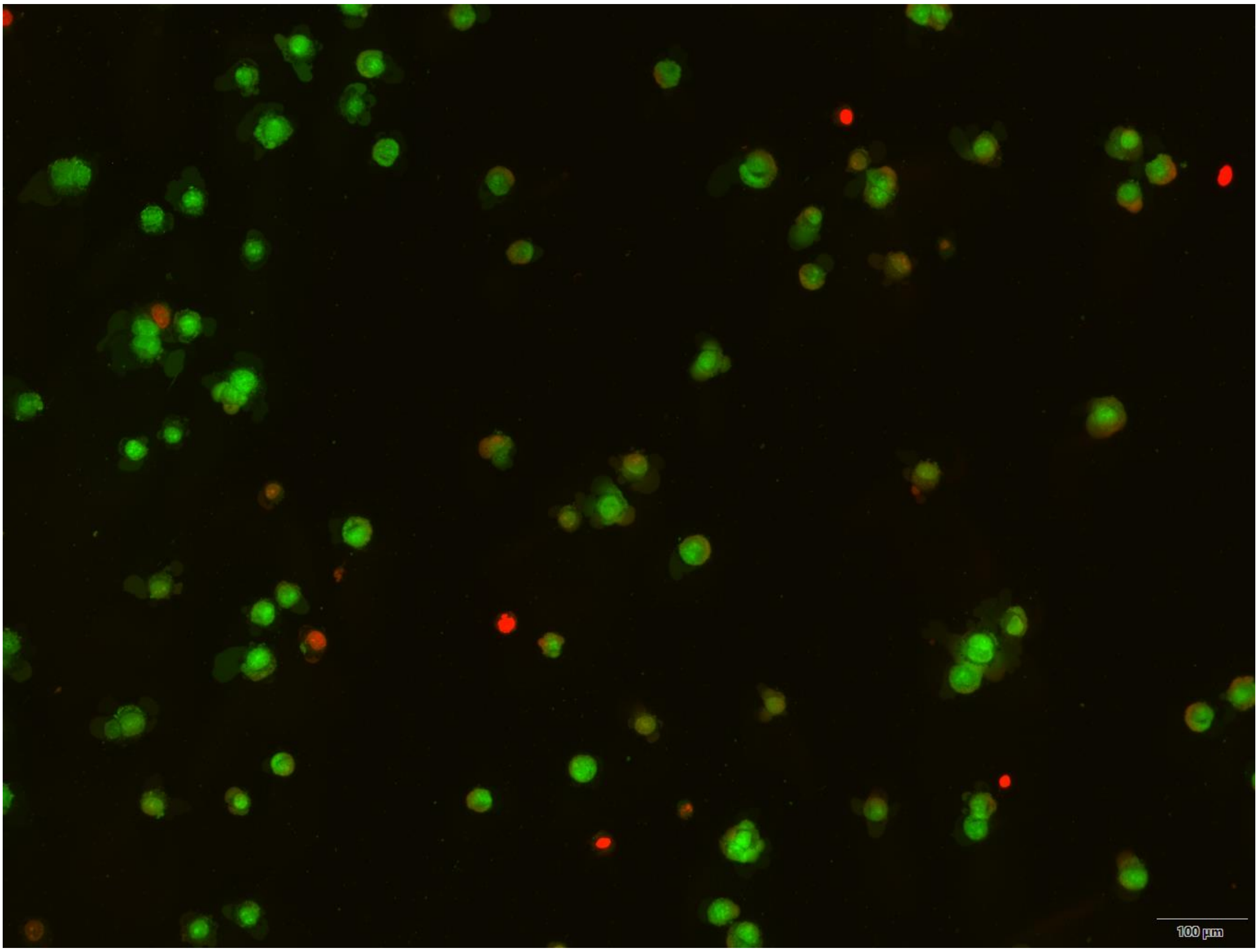

Merge

(B) IGF-1 group

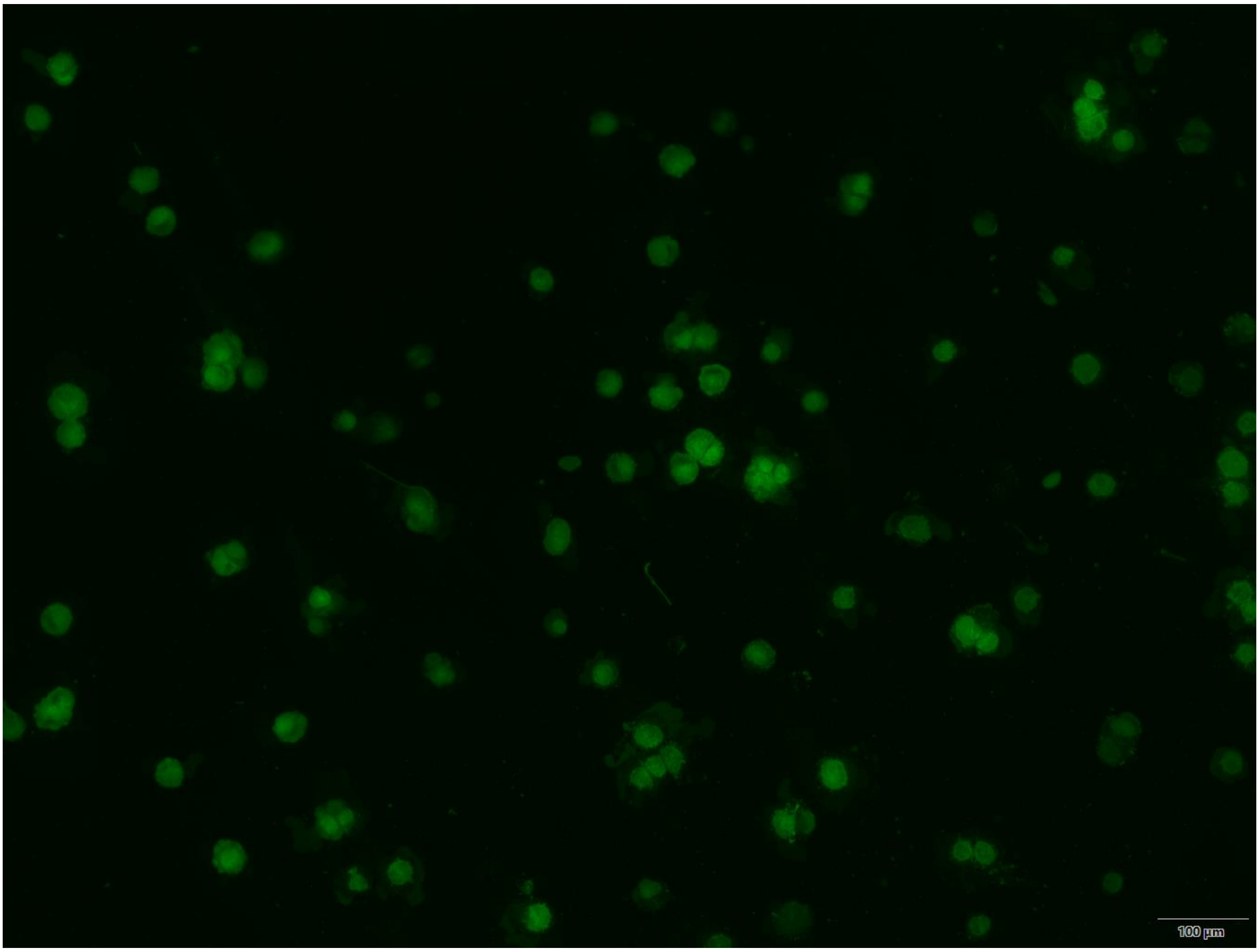

AO staining

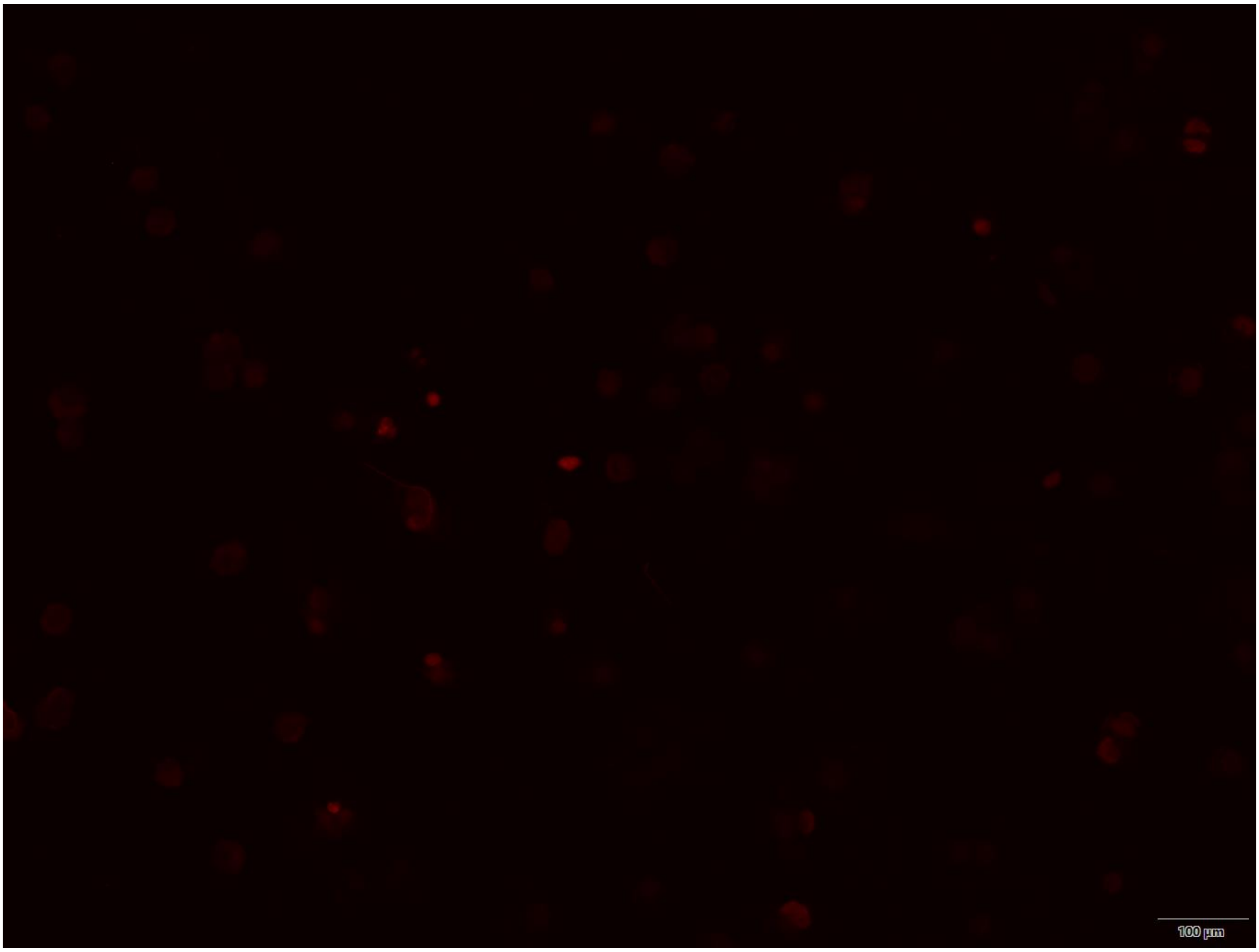

EB staining

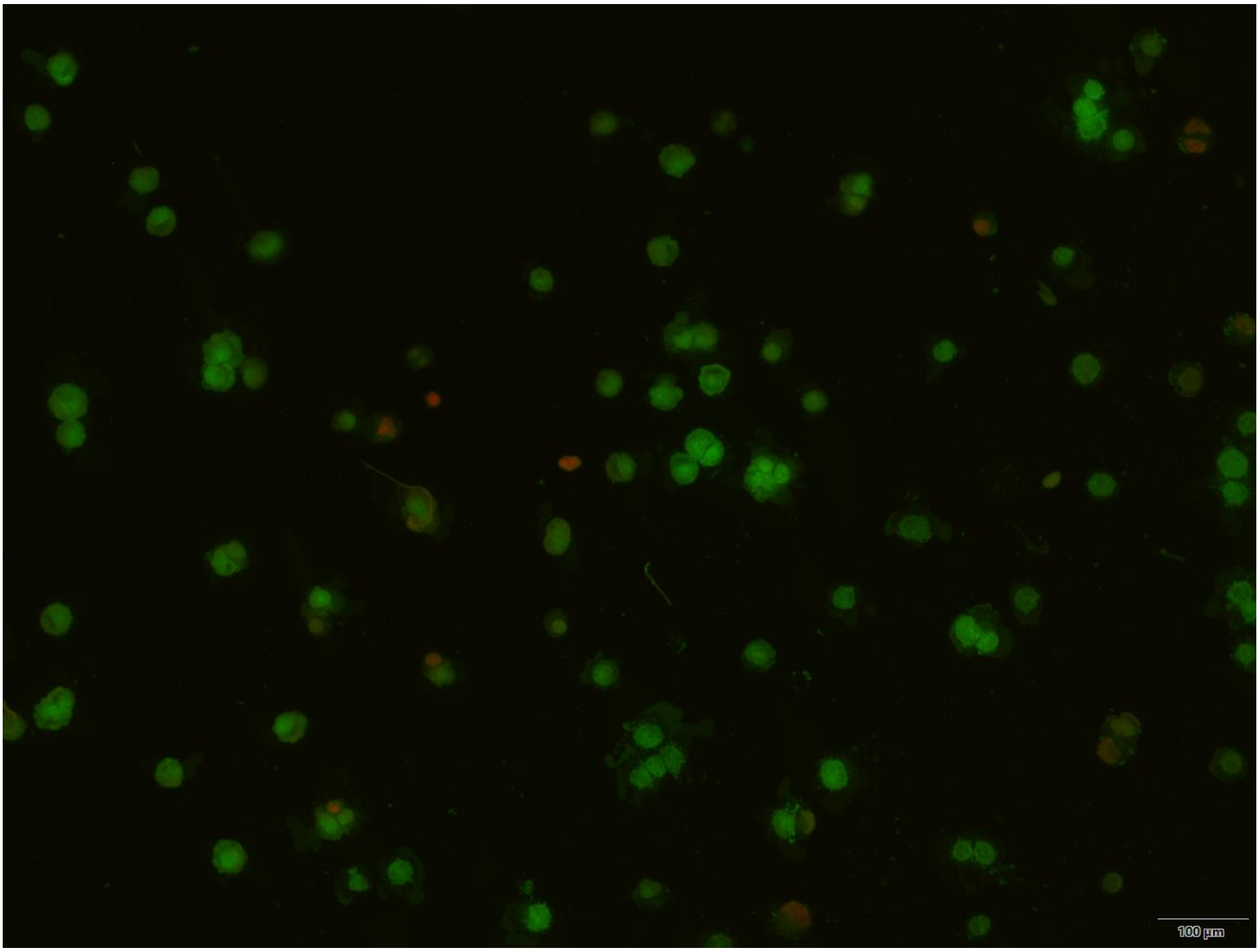

Merge

(C) Pae group

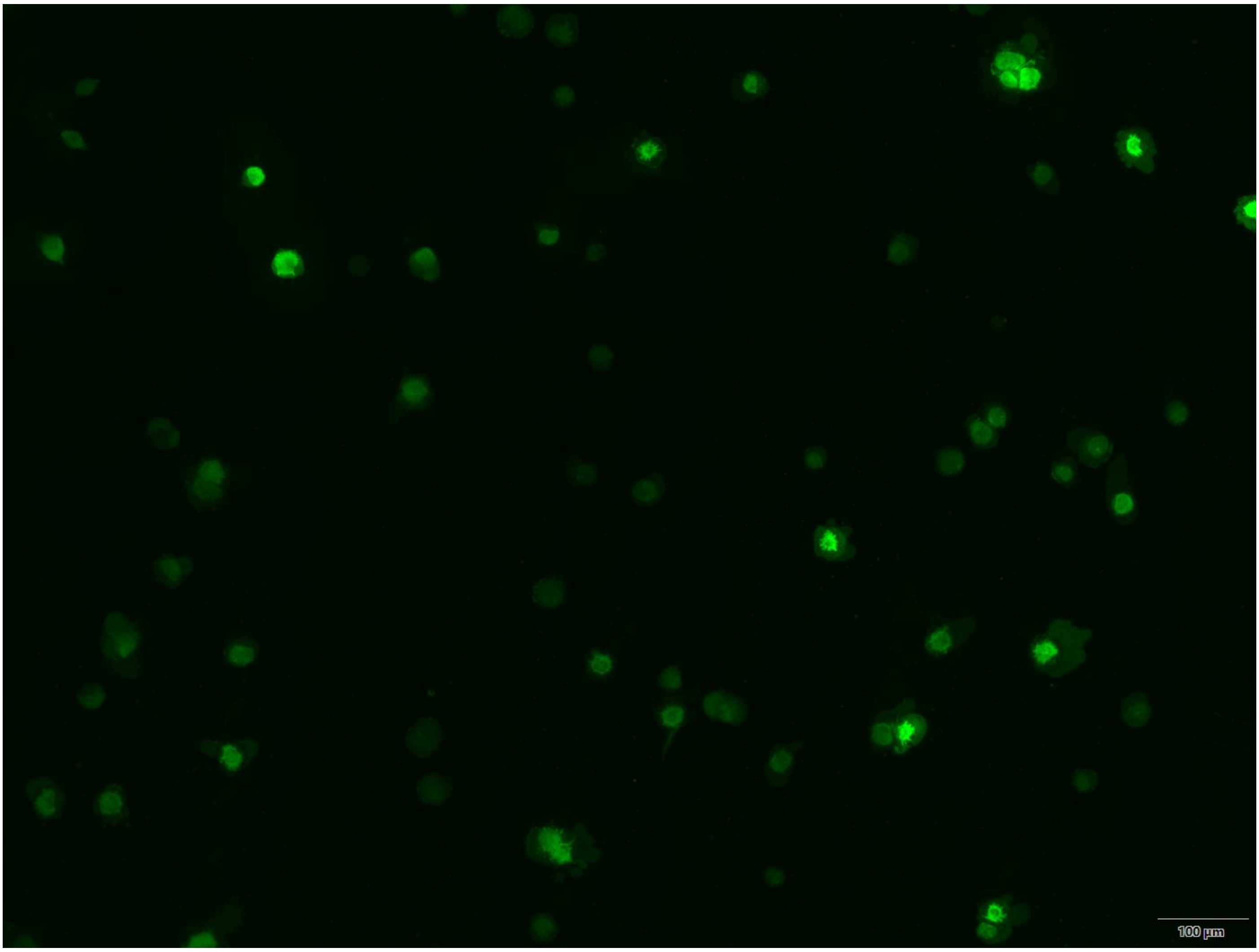

AO staining

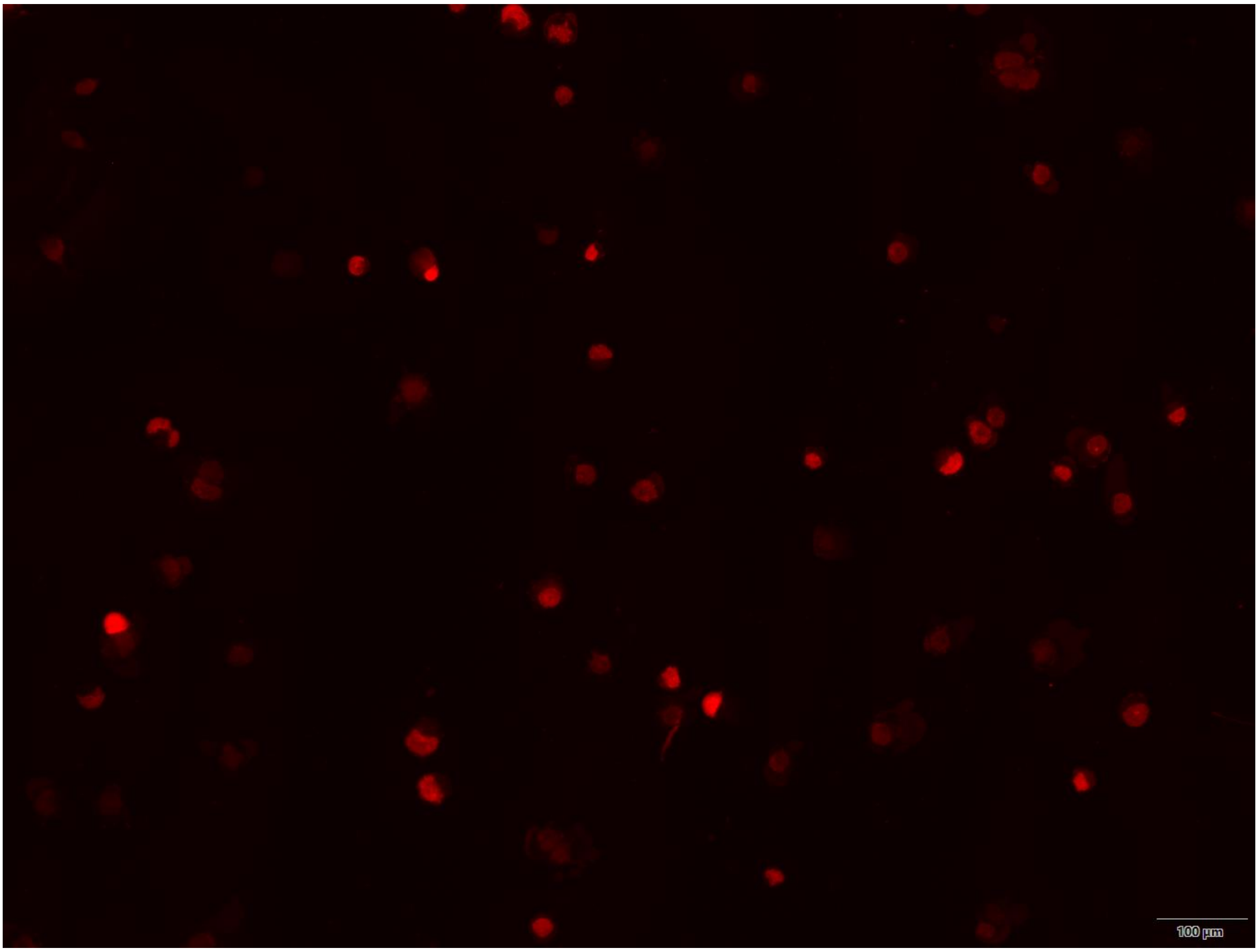

EB staining

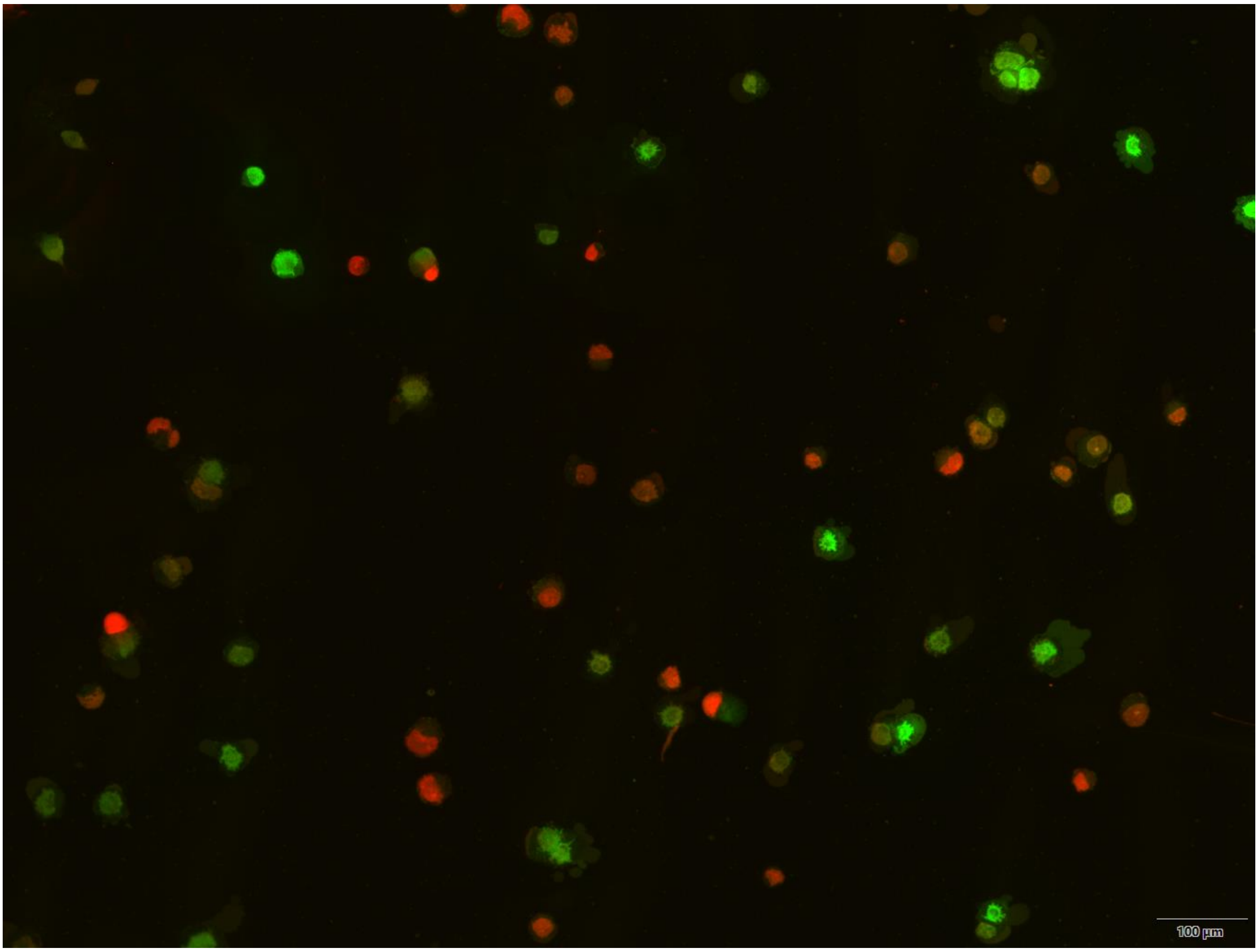

Merge

(D) IGF-1+Pae

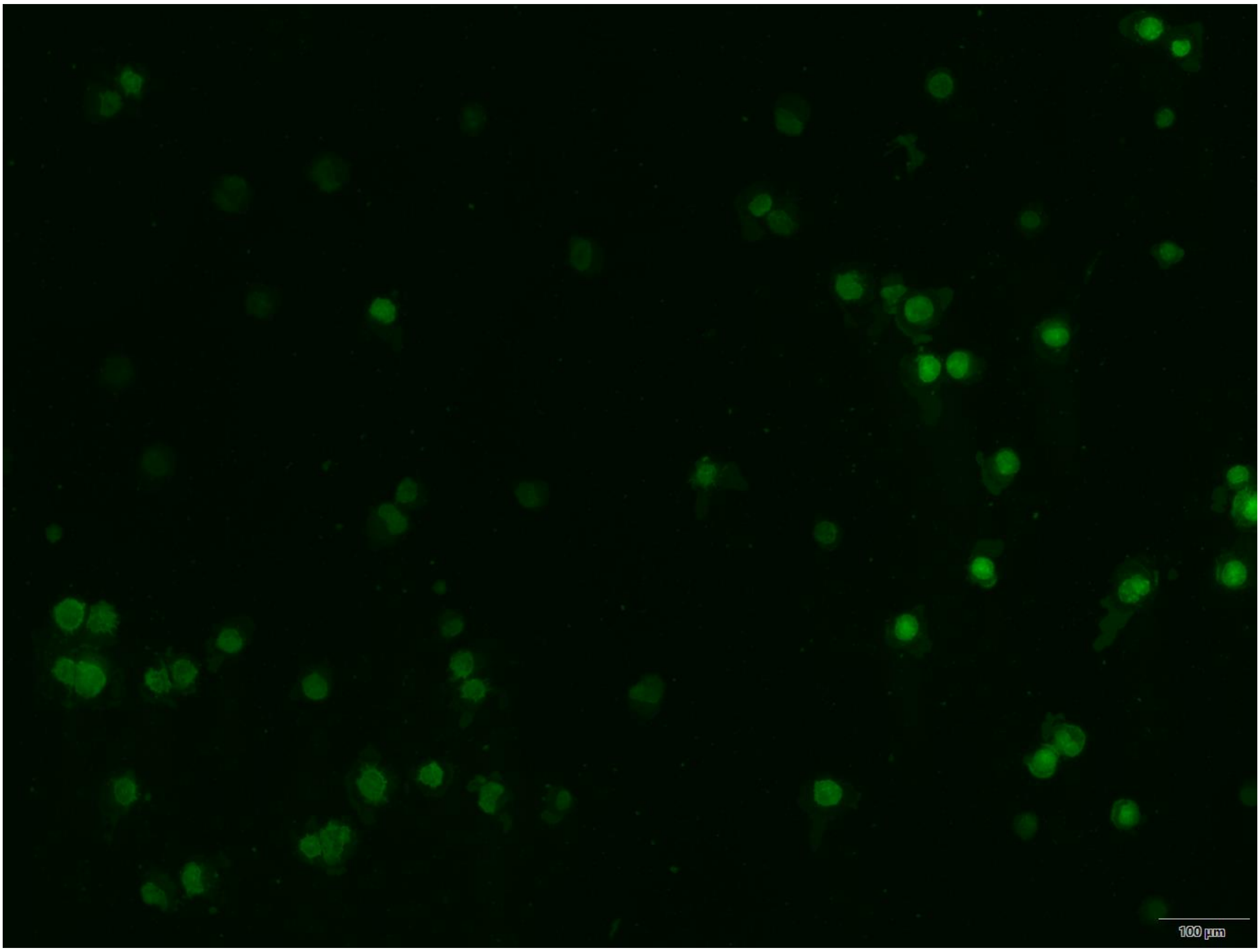

AO staining

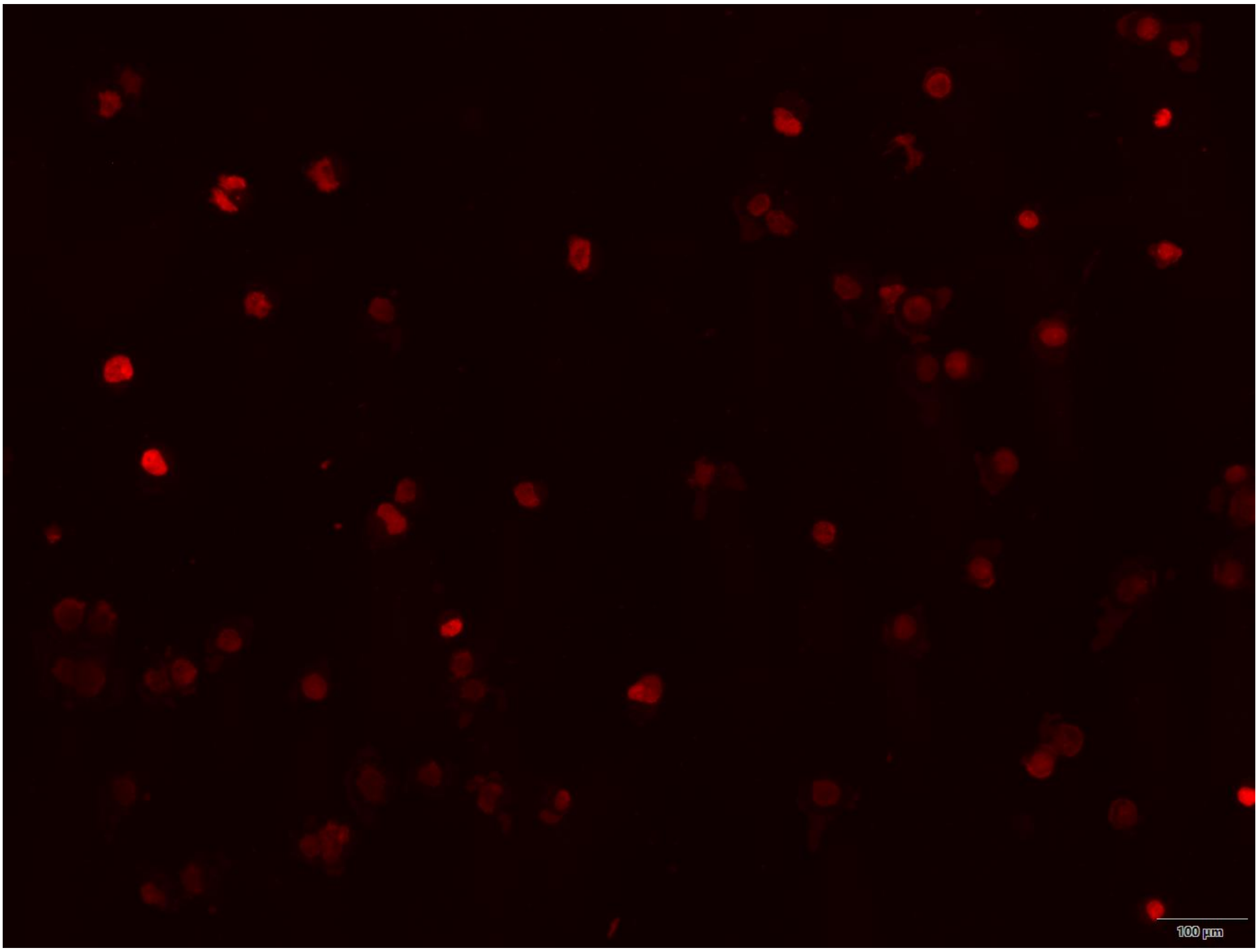

EB staining

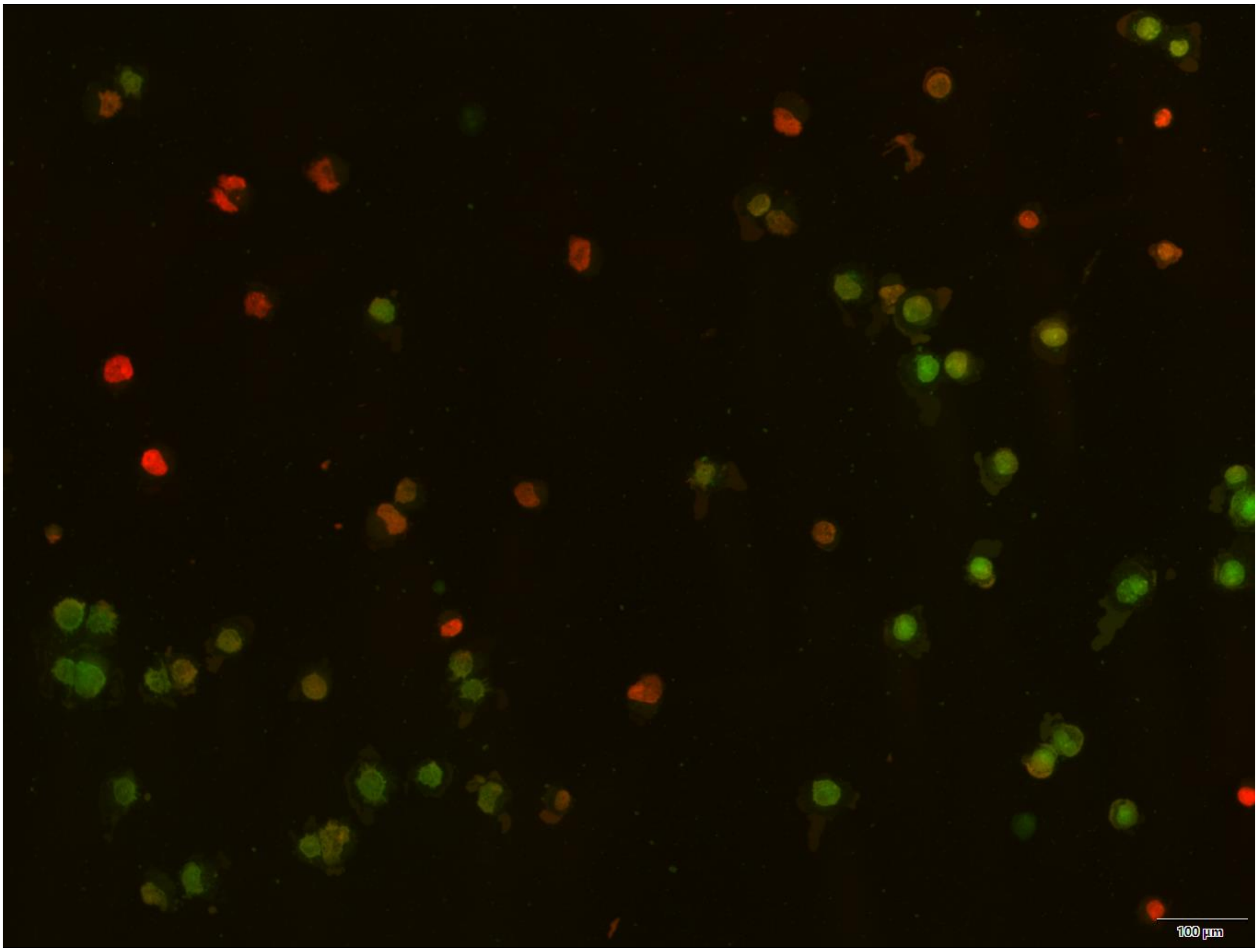

Merge

Supplementary Figure S4. (A and B) Uncropped blots for FoxO1 and β-actin in Fig. 4A.

Groups: Ctrl, IGF-1, Pae, IGF-1+Pae (From left to right)

(A) FoxO1 blotting

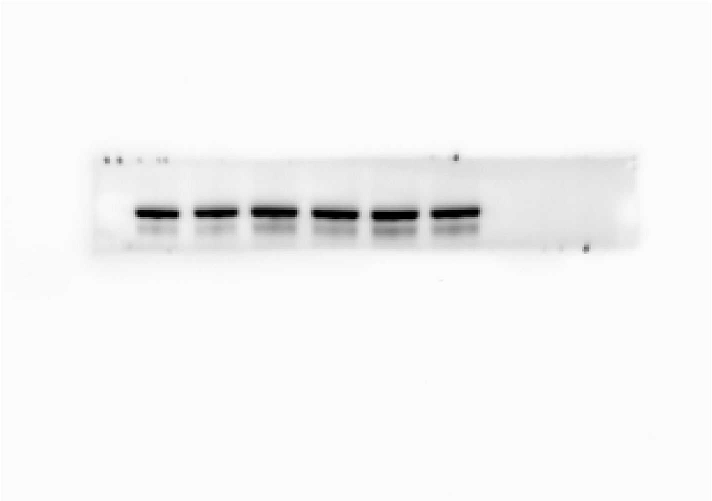

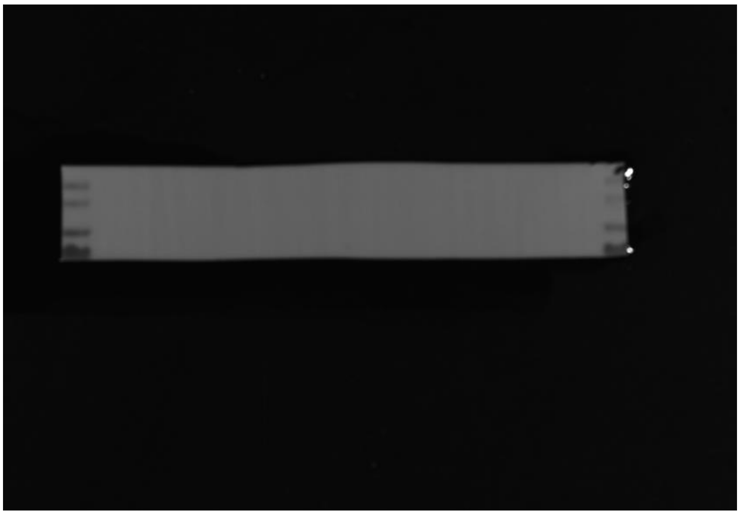

(B) β-actin

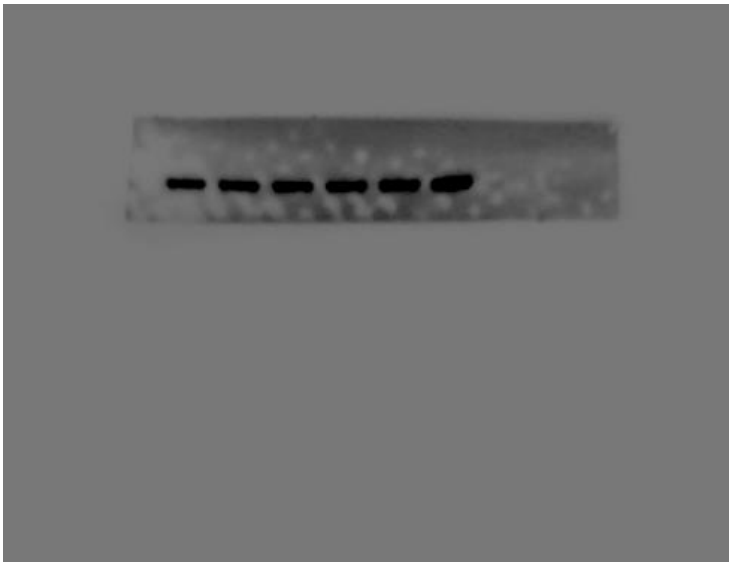

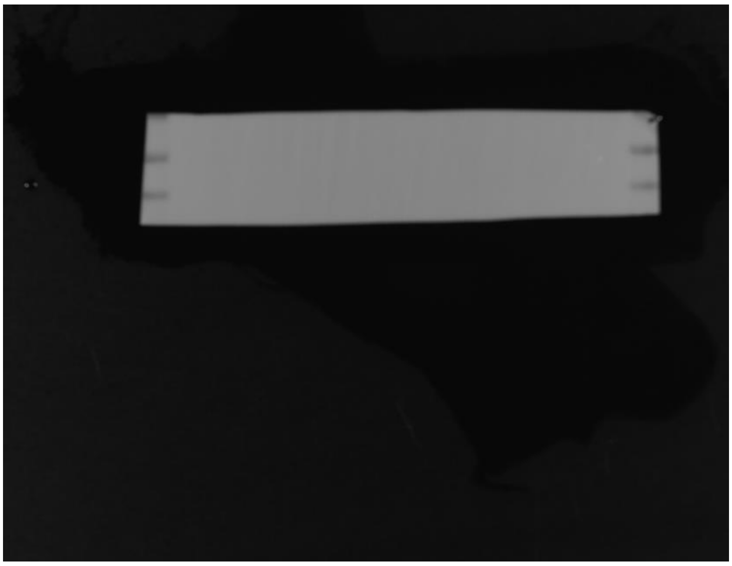

Supplement: Supplementary file 1 — Supplementary material 1. [file 13659_2024_478_MOESM1_ESM.pdf]
